# Typhoid Fever, Part 2

**Published:** 1897

**Authors:** Constantine Hering


action: *Coccul.*, as if from pain: *Stramon.*; stupid expression: *Hyosc.*; sunken features: *Zincum*; anxious, hippocratic —: *Arsen.*; expression of fear and terror: *Stramon.*; wild expression of features with fullness and redness of face: *Bellad.*

**Cheeks**; yellow, with central deep flush: *Baptis.*; — reddened: *Bellad. Hyosc. Mur. ac. Stramon.*, and hot: *Bellad. Rhus tox.*; — red and burning: *Bryon.*; — burning hot, with circumscribed redness: *Arsen. Mur. ac.*, in afternoon: *Lycop.*, with delirium: *Lachnanth.*; one — red, the other pale: *Chamom.*; alternately red and pale: *Veratr.*, — red, even blue: *Bellad.*

**Face**; dark red with snoring: *Opium*, with a besotted expression: *Baptis.*; — red, with heat: *Veratr.*; — red and cracked: *Zincum*; — red and bloated: *Opium*; — blue: *Carb. veg. Laches. Veratr.*; — bluish red, with feeble intermitting pulse: *Veratr.*; — brown, bluish, black: | *Arsen.*; — black and cracked: *Laches.*; — shining and puffed up: *Bellad.*; — red and bloated: *Opium*; pale, waxy complexion: *Zincum*; — sunken and pale, with pointed nose: *Coccul.*; — waxy, white or yellow: *Silic. Zincum*; — yellow: *Lycop.*, and dirty color: *Mecur.*; — yellow and pale: *Bryon.*; — suddenly yellow and pale, after the first week (generally fatal): *Lycop.*; — pale; sickly aspect: *Kali carb.*, as after long illness; with great uneasiness, and sunken eyes, with blue circles around them: *Sulphur*; sick look, mostly in the evening; hollow, sunken eyes, with blue circles: *Phosphor.*; — pale, ashy: | *Phosphor.*; ashy color with wild delirium, alternating with restlessness, wants to be somewhere else; dry tongue, smoky nose, diarrhœa, prostration, subsultus tendinum and deafness: *Chlorum*; — pale, dingy, or earth-colored, and sunken: *Arsen. Phosphor.*, and cold: *Carb. veg.*; — collapsed: *Camphor*; — sunken: *Colchic. Laches. Mercur. Zincum*; — pale, and expression dull: *Secale*; nose pointed, eyes sunken: *Coccul. Phosph. ac.*; — yellowish and sunken: *Lycop.*, with sharp nose, and hollow eyes, with dark circles around them, with insensibility and indifference: *Cinchon.*; — and eyes sunken: *Cinchon. Mercur. Phos. ac. Phosphor. Sulphur*; — pale, cold, cadaverous, sharpened nose and sunken cheeks, blue circles around the eyes: *Veratr. alb.*; deathly appearance of —: *Mercur.*; hippocratic —: *Arsen. Carb. veg. Cinchon. Colchic. Phosphor. Secal. Zincum*; cadaverous aspect, and extreme prostration: *Colchic. Veratr.*, while heat of body is retained: *Mercur.*; petechiæ on —: *Bellad.*

**Jaw**; rigidity of the muscles: *Baptis.*; pain in joint of lower —: *Baptis.*; — falling: *Arsen.*; — dropping: *Baptis. Laches.*; — hanging down: *Laches. Lycop. Mur. ac. Opium.*; — hanging during the coma: *Laches.*; — sunken: *Secale*; lower lips hanging: *Opium*; trembling: *Arnic.*

### OUTER MOUTH.

Mouth open: *Mercur. Phosphor.*, to the widest extent: *Colchic.*; distortions: *Bellad. Camphor. Opium.*

**Lips**; trembling: *Stramon. Sulphur*; — and tongue pale: *Phosph. ac.*; — bright red: *Sulphur*; — dark red, and dry: *Bellad.*; — bluish red, black or brown:

*Arsen.*; — dry and brown: *Nitr. sp. d.*; — covered with brown slime: *Colchic. Arsen.*; crusts on the —: *Arsen.*; — blue: *Carb. veg. Cinchon.*; — dry and blackish: *Arsen. Bellad. Bryon. Laches. Phosph. ac. Rhus tox. Veratr.*; — coverêd with black slime: *Arsen. Cinchon. Phosphor.*; — as if parched by thirst: *Arnic.*, with dryness of the palate: *Phosphor.*, and brown or black: *Rhus tox.*; crusts on — and tongue: *Arsen.*; —, gums, and teeth covered with brown or black slime: *Arsen.*; —, teeth, and tongue covered with a thick, brown coating: *Colchic.*; — dry, parched and covered with brown or black crusts: *Arsen. Rhus tox.*; — dry: *Mur. ac.*, and cracked: *Arsen. Bellad.*, and brown: *Bryon.*, and hard: *China*; — cracked: *Colchic. Pulsat. Sulphur*, and sore: *Stramon.*; — dry and black: *Veratr.*, and bleeding: *Laches.*

**Corners of mouth**, sore: *Arsen. Arum triph.*; pain as if cut; excoriation and cracks, ulceration, with pains as of excoriation: *Mercur.*; — and upper lip raw and sore from acrid ichorious discharge from nose: *Arum triph.*; eruption around lips: *Phosph. ac.*

**Teeth**; grinding: *Arsen. Colchic.*; grating: *Hyosc.*, — covered with brown or black slime: *Arsen.*; sordes on —: *Baptis.*, and lips: *Stramon.*; — covered with a thick brown coating: *Colchic.*, brown slime: *Hyosc.*; — covered with a dark mass: *Hyosc.*, brown mucus, deposited on —: *Sulphur.*

**Gums**; at the beginning, swollen and bleeding: *Mercur.*; — spongy, separating and bleeding on the slightest touch: *Carb. veg. Mercur. Phosphor.*; — and teeth covered with brown or black slime: *Arsen.*; — covered with brown mucus: *Rhus tox.*

**Taste**; and smell very acute: *Mur. ac.*; — insipid, pappy, and slimy: *Mercur.*; pappy — in the mouth: *Coccul.*; — bad: *Baptis.*, foul, nauseous, viscous, flabby, soap-like: *Mercur.*; food is without taste: *Nux vom.*; tastes neither food nor drink: *Rhus tox.*; food tastes like straw: *Stramon.*, like nothing: *Arsen.*; — bitter: *Bryon. Laches.*, of things eaten: *Pulsat.*

**Speech**; stammering: *Arsen. Bellad. Carb. veg. Lycop. Secal. Stramon.*; — as if the tongue were too heavy, stuttering: *Veratr.*; unintelligible —: *Arsen.*; — lisping and stammering: *Bellad. Opium. Stramon. Veratr.*; — muttering and mumbling; it costs him great effort to speak the words plainly: *Coccul.*; impeded —: *Phosph. ac.*, from a dry, hard glazed tongue: *Arsen.*; falters and hesitates in speaking, aversion to the effort: *Sulphur*; only answers with indignation: *Pulsat.*; — difficult, weak, indistinct: *Secal.*; indistinct —: *Hyosc.*; nasal —: *Laches.*, from dryness in the throat: *Byron.*; speaks slow and weak as if the organs had an impediment to overcome: *Secal.*; as if they could not move the tongue: *Carb. veg.*; reluctant —: *Bellad.*; — difficult: *Phosph. ac.*, with heaviness of the tongue: *Laches.*; — from difficult respiration, and great debility: *Bellad.*; can only speak loud with great effort: *Opium*; inability to speak loud: *Nux vom.*; lisping: *Arsen. Bellad. Stramon.*: loss of —: *Colchic. Hyosc. Veratr.*, and of consciousness: *Mercur.*: with subsultus: *Secal.*; organs as if paralyzed with lisping and stammering: *Stramon.*; complete aphasia: *Bellad.*; inability to talk and to put out the tongue, which is cracked, sore, and ulcerated: *Apis.*; hasty —: see Mind.

**Tongue**; trembling and hard to put out: *Gelsem. Laches.*; — trembles when put out: *Bellad. Laches. Secal.*; — trembling: *Arsen.* ‖*Bellad. Gelsem.* ‖*Laches. Lycop. Secal.*, when protruding: *Laches. Stramon.*, and dry and cracked: *Arsen.*; — sometimes spasmodically thrust to and fro between the teeth: *Lycop.*; — comes out dry and black with a snapping noise, and goes like a pendulum: *Lycop.*; — trembling in the attempt of protruding: *Secal.*, or the tip remains under the lower teeth, or lip, and does not come out: *Laches.*; can hardly put the — out, it trembles: *Gelsem.*; cannot put the — out: *Gelsem, Hyosc. Laches.*, or if he does, it trembles: *Lycop.*; after the — is put out with difficulty, can hardly draw it in: | *Hyosc.*; — protruded with difficulty: *Colchic. Laches.*; — does not move rightly: | *Bellad.*; cannot move the — at will, even if conscious: *Mur.* ac.; — stiff, like a piece of wood: *Arsen.*; — heavy, stiff and numb: *Colchic.*; — as if stiff, difficult motion while swallowing: *Laches.*; — immovable from dry black crusts: *Phosphor.*; sensation as if — was too thick, while speaking: *Nux vom.*; — heavy, with stammering as if drunk: *Bellad. Laches.*, and immobility, with difficult speech, scarcely movable: *Carb. veg.*; paralytic weakness of —: *Bellad.*; — paralyzed: *Baryt. c. Hyosc. Laches.* ‖ *Mur. ac. Opium. Stramon.*; — paralyzed on the right side (turns to the left): *Bellad.*; — as if burnt, and insensible, coated: *Pulsat.*

Tongue dry: *Bellad. Chlorum. Ginseng. Hyosc. Lachnanth. Laches.* | *Mur. ac. Natr. mur. Nux mosch. Opium. Phosphor. Phosph. ac. Stramon. Veratr.*; — and lips dry: *Phosph. ac.* See Lips. Very dry —, nose, and lips: ‖ *Phosphor.*; burning — and lips; looks like burnt leather: *Hyosc.*; — rattles like leather: *Mur. ac.*; — dry and hard: *Mercur.*, and glazed: *Arsen.*, and stiff as a piece of board: *Arsen.*, and cracked: *Bellad. Hyosc.*, and red: *Laches.*, and trembling: *Arsen.*; great sensation of dryness of —, with excessive thirst, drinks but little at a time: *Arsen.*, and great thirst, drinking much: *Bryon.*; — not coated, with desire for drink: *Rhus tox.*; dryness of — without thirst: *Opium. Pallad.*; — as if burnt, and yet no thirst: *Magn. mur. Pulsat.*; — dry, with diarrhœa: *Phosphor.*, and debility: *Natr. mur.*, in the morning: *Sulphur*; — red: *Hyosc. Lachnanth. Rhus tox.*; —, bright red: *Colchic.*; — covered all over with raised papillæ: *Ant. tart. Bellad.*, and dry: *Arsen. Baptis. Bellad. Laches. Lycop. Rhus tox.*; — dry and cracked: *Arsen. Hyosc.*, and hard: *Hyosc. Rhus tox.*, and hot: *Bellad.*, and glistening: *Kali bichr. Laches.*; — dry and dark red: *Baptis.*; — raw, painful, inflamed in middle: *Gelsem.*

Tongue clean, or only lightly coated: *Gelsem. Mur. ac.*, with gastric and other derangements: *Cina. Digit.*; — clean and dry: *Stramon.*; — dry, with thirst: *Rhus tox.*; — dry, red, and hard: *Bryon. Hyosc.*; — almost clean, with hard, bright red edges, and cracked lips: *Opium.*

Tongue smooth, not very dry: *Phosph. ac.*, and white: *Bellad.*, and glossy as if deprived of its papillæ: *Terebinth.*

Tongue rough and dry: *Stramon.*, and cracked, and dark brown: *Bryon.*; — brown or blackish: *Mercur.*;

— sticks to the roof of mouth: *Nux mosch.*; — covered with a thick tenacious paste, like a layer of putty spread over it: *Bryon.*

Tongue brown: *Baptis. Hyosc.*; — colored brown and black: *Secale*, and dry: *Hyosc.*, and hard: *Rhus tox.*, and parched, as hard as a board, with diarrhœa: *Rhus tox.*; crusts on — and lips: *Arsen.* See Lips. — brownish: *Nux vom.*; — pale: *Carb. veg.*; — lead-colored: *Arsen. Carb. veg.*; — bluish, or pale: *Carb. veg.*, and small: *Mur. ac.*; — blue, pale or sticky: *Carb. veg.*; dark colored —: *Bryon. Staphis.*; blackish —: *Mercur. Veratr.*; — black: *Arsen. Laches. Opium. Rhus tox. Secal.*, and cracked: *Cinchon. Veratr.*, with deep edges: *Nux vom.*; — raw and burned: *Cinchon.*, and dry: *Arsen. Laches. Lycop. Opium. Phosphor. Rhus tox.*; — furred very lightly: *Mur. ac.*; — and mouth covered with a muddy paste: *Carb. veg.*; hard thick phlegm on —: *Rhus tox.*; — dry, brown, as if burned: *Arsen. Lycop.*; — full of thick dirt, or hardened phlegm: *Rhus tox.*; thick and gray coating on —: *Mercur.*; grayish-white slime on —: *Phosph. ac.*; — as if coated with a skin: *Phosphor.*; — sticky and dirty: *Lycop.*, and moist: *Carb. veg.*, and yellow: *Pulsat.*; thick white, or dirty white coating on —: *Bryon.*; — furred in the beginning: *Coccul.*; thick dirty-white coating on —: *Mercur.*, and stools of greenish mucus: *Nitr. ac.*; white coating with sore spots on —: *Nitr. ac.*; — has a red margin and white centre: *Bellad. Gelsem. Sulphur*; — furred in the middle and behind, not on edges: *Bryon.*; — dry, with a brown streak in the middle: *Arnic. Baptis.*; two white stripes on a red ground: *Bellad.*; — yellowish-brown, dry in centre: *Stramon.*; white coating on sides of —: *Caust.*, in centre: *Bellad. Bryon. Gelsem. Phosphor. Sulphur*; sore spots on —: *Nitr. ac. Tarax*; mapped —: *Arsen. Lycop.* | *Natr. mur.* || *Tarax.*; — yellow: *Bryon.*, and sticky: *Pulsat.*, and rough, or dry, with whitish-yellow coating: *Baptis. Coccul.*; — yellow and red: *Pulsat.*, yellowish, later brown and dry: *Bryon.*; gray coating, and cracked: *Pulsat.*; — brown: *Hyosc.*, and dry; mostly in the centre: *Baptis.*; — grayish-white: *Phosph. ac.*; — thickly covered: *Colchic.*, and cracked: *Bryon.*; brownish, cracked crusts on —: *Phosph. ac.*; — covered with dark brown sordes of an offensive odor; foul —, covered with a thick layer of yellowish or brownish fur: *Bryon.*: remaining after Bryonia: *Nux vom.*

Tongue cracked: *Apis*, and parched: *Carb. veg.*; — as if scalded: *Veratr. vir.*; — as if burnt: *Arsen.*, and rough, dry, and dark brown: *Bryon.*; — thick: *Nux vom.*, and too red, dry, and swollen: *Veratr. alb.*; — swollen and dry: *Stramon.*; small atrophied —; substance altered: *Mur. ac.*; — indented from the teeth: *Ant. tart.*; soft — with imprints of the teeth: *Kali hydr. Mercur. Rhus tox. Stramon.*; shooting in the —: *Bryon.*; — red, raw, painful and inflamed in the middle: *Gelsem.*; — sore, red, with elevated papillæ: *Arum triph.*; — painful, as if chapped and burning: *Mercur*; — covered with black crusts: *Phosphor.*, with vesicles: *Apis*; white coated — with sore spots: *Nitr. ac.*; — has a coating, which on removal, leaves sore spots: *Tarax*; — cracked, sore, and ulcerated: *Apis. Baptis.*, fissured, dry: *Carb. veg.*

Tip of tongue remains under lower teeth, or lip, when attempting to put it out: *Laches.*; clean and pointed —: *Kali carb.*; heat and dryness of —: *Carb. veg.*; — dry: | *Nux vom.*, and red: *Bellad.* (*Rhus tox.*); undefined redness of —, and redness of the borders: *Ant. tart. Bellad. Rhus tox. Sulphur*; — red, in the shape of a triangle: *Rhus tox.*; a whitish coating on one side: *Rhus tox.*, on both sides: *Caust.*; — red and cracked, or cracked and bleeding: *Laches.*; — as if burnt and rough: *Phosphor.*; fissures on the —: *Laches.*

**Dryness**; of mouth: *Chlorum.*, and tongue: | *Mur. ac.*, and food tastes like straw: *Stramon.*; — in fore-part of mouth, and on tip of the tongue in early stage: *Nux vom.*; — of mouth and fauces: *Phosph. ac.*; — from the fauces into the nose: *Bellad.*; — of mouth and throat: *Magn. mur. Nux mosch. Nux vom. Phosph. ac. Pulsat.*, at night: *Coccul.*, in the morning, as if from the use of spirituous drinks the evening before: *Nux vom.*; sense of — or actual —, afternoons and after midnight: *Rhus tox.*; — and thirst: *Arnic. Baptis. Bryon. Laches. Rhus tox. Secal. Veratr.*; severe —, and not relieved by drinking: *Phosphor. Rhus tox.*, drinks large quantities at a time: *Arnic. Bryon.*; — without thirst: *Bryon. Magn. mur. Nux mosch. Pulsat.*; — but not much thirst, water does not relieve: | *Phosphor*; feeling of —, or actual — without thirst: *Bryon.*, but has to moisten the mouth: *Stramon*; — of mouth and throat: *Magn. mur. Pulsat.*; — in the evening, so great, that the tongue sticks to roof of the mouth, yet without thirst: *Nux mosch.*

**Mouth**; sticky, no thirst nor appetite: *Gelsem.*; — intolerably sticky: *Phosphor.*; — filled with a slimy mucus: *Mercur*; accumulation of frothy, soap-like saliva in — and throat, almost choking: *Bryon.*; — full of thick viscid saliva: *Baptis.*; salivation: *Hyosc.*; buccal cavity raw and sore: *Arum triph.*; hemorrhage from nose and —: *Carb. veg.*; raw — and throat: *Arum triph.*; the whole inside as if raw: *Stramon.*; aphthæ: *Baptis.*; small and bluish, with putrid acid stench: *Mur. ac.*, putrid: *Nitr. ac.*, sour: *Sulph. ac.*; coma: *Camphor. Helleb.*

**Smell**; offensive —, morning and evening: *Hyosc.*, and tongue very dry in the morning: *Sulphur*; metallic odor: *Mercur*; fetid breath: *Arsen.* | *Arnic. Baptis. Mercur.*; putrid odor: *Arnic. Arum triph.*, morning and night: foul smelling slime covers the tongue, in the morning on waking, with dryness of mouth and throat: *Pulsat.*; — putrid, like carrion: *Nux vom.*; cadaverous —: *Hyosc.*

### THROAT.

Gurgling when drinking; the fluid rolls audibly down into the stomach: *Arsen.*; the drink rolls audibly down the throat, as though it were poured into an empty barrel: *Hydr. ac.* (*Coccul.*); the fluid rolls audibly down the throat, and stools and urine pass off involuntarily: *Moschus*; spasms of the pharynx: *Bellad.*; choking sensation from the stomach up into the throat, with oppression in the chest, better from belching: *Ignat.*; chokes even with half a spoonful of water; inability to swallow: *Baptis.*; difficulty in swallowing liquids: *Bellad.*; difficulty in swallowing: *Apis*; swallowing very slow: *Helleb.*; complete inability to swallow: *Stramon.*; intense burning in throat: *Veratr. vir.*

**Dryness**: *Bryon. Nux mosch. Secale*; — hardly permits swallowing: *Phosphor.*; — in throat and on tongue, without thirst: *Pallad.*; — of palate: *Phosphor.*; — only of the palate: *Stramon.* For mucus and phlegm, see Larynx.

**Sore throat**: *Baptis.*; — with deafness: *Laches.*; chewing or swallowing food, or drinking causes suffering from the raw mouth: *Arum triph.*; efforts to swallow produce violent pain; tonsils enlarged; uvula inflamed and elongated: *Baptis.*; swollen glands of throat and neck: *Arum triph.*; angina faucium: *Mercur.*

**Appetite**; too great during reconvalescence: *Pulsat.*; hunger in convalescence, commences to eat, but does not enjoy it, everything is so bitter: *Pulsat.*; when the — will not return in convalescence: *Sulphur. Psorin.*; loss of —: *Bryon. Nux mosch.*, with fullness of the stomach: *Nux mosch.*; loss of —, and prostration: *Rhus tox.*; would not eat nor drink, but without any nausea: *Iris vers.*; repugnance to all ingesta, tastes neither food nor drink: *Rhus tox.*; in a hasty way, they push back the food they wanted: | *Bellad.*; asks for food, but refuses it when offered: *Phosphor.*

**Thirst**: *Bellad. Hyosc. Mercur*; much —: *Hydr. ac.*; great —: *Carb. veg. Mur. ac. Stramon.*; unquenchable —: *Natr. mur.*; inextinguishable —: *Colchic.*, with a red, dry tongue: *Camphor*; does not want anything but drink: *Opium*; — with a dry tongue: *Bryon. Rhus tox.*; — not relieved by drink: *Rhus tox.*; constant — for water: *Phosphor.*; — day and night, for cold drinks, especially for water; *Mercur.*; — for cold water: *Arsen.*; — for cold drinks: *Bryon.*; — with dry lips, and a bright red tongue: || *Bellad.*; — afternoon and evening, with cold sweat: *Veratr.*; continual —, wherein the patient only wets his lips and cannot drink much: || *Arsen.* H.; water molests the stomach: *Sulphur*; water does not taste well: *Natr. mur.*, is not pleasant: *Calcar. Sambuc.*; burning — but does not drink much: *Bellad.*; spitting out of liquids put into the mouth: *Baptis.*; continual —, but only wets lips, cannot drink much, every swallow becomes disgusting: *Arsen. Lycop.*; violent — day and night, does not drink often, but much at a time: *Bryon.*, large quantities: *Hyosc.*; does not ask for water, but when offered takes it hastily: *Opium*; hasty drinking: *Hepar s. c.*; — with nausea or vomiting: *Arsen.*; continual — with nausea: *Natr. mur.*; — mostly in the evening, with aversion to water: *Nux vom.*; water does not taste well: *Natr. mur.*; — with heat, from 3 to 6 P. M.: *Phosphor.*; inextinguishable —, trunk hot and extremities cold: *Colchic.*, burning in head and coldness of trunk: *Arnic.*

**Thirstlessness**; with dry tongue: *Magn. mur. Opium. Pallad.* | *Pulsat.*; — with dry mouth: *Nux mosch.*; no desire to drink, yet much burning heat: *Hyosc.*

Wants acids: *Arsen. Bryon.*, alcoholic drink: *Arsen.*, wine: *Bryon. Coffea*; desire for refreshing acid things, fruit, wine, white-beer: *Mercur.*

**Eating**; after —, sour taste: | *Nux vom.*; diarrhœa immediately after —: *Phosphor.*; great chilliness after taking anything to eat, or drink: *Tarax*; after —, chilliness: *Arsen. Arum triph. Calc. ostr. Carb. veg. Caust. Ipec. Kali carb. Nux vom.* || *Tarax. Zincum.*

**Drinking**; hurts in the throat: *Hydr. ac.*; water molests stomach: *Arsen. Phosphor. Sulphur*; after — the fluid makes a rumbling noise in the abdomen, as in an empty barrel: *Hydr. ac.*; after —, immediately distention and pain in bowels: *Nux vom.*; after — rumbling: *Mercur.*; — with haste, rumbling in bowels: *Arsen.*; after — water, abdomen bloated and sensitive: *Mancin.*; after —, cold fingers: | *Tarax.*

### GASTRIC SYMPTOMS.

**Hiccough**: *Arsen. Nux mosch.*; with sighing: *Secale.*

**Belching**: *Coccul.*; empty —: *Bryon.*

Struggles with frequent squeamishness: *Arsen. Bryon. Calc. ostr. Lycop. Phosphor.*

**Nausea**: *Coccul.*; retching: *Bryon.*; — and vomiting with painful sensibility of epigastrium: || *Bryon.*

**Vomiting**: *Bryon.*; — and retching: *Arsen.*; — lessened by swallowing water, but when it gets warm in the stomach, it comes up again: | *Phosphor.*; — water with sour taste: | *Phosphor.*; — food and other things: *Calc. ostr.*; — phlegm and food: | *Veratr.*; — water and green slimy fluid, not that which was eaten: *Arsen.*; — watery, bilious, and slimy masses with great pain: *Phosphor.*; — thick, black bile mixed with tenacious, bilious mucus, as if decomposed; a quart at a time: *Secal.*; — after bile and mucus, black bile and blood: *Veratr.*; — when moving: ‖ *Bryon.* ‖ *Sulphur*; — and watery diarrhœa: *Veratr.*; — with burning heat: *Chamom.*; — after delirium: *Secal.*; — with headache: *Kali carb.*

**Scrobiculus**; sinking, weak feeling in —: *Ignat.*; great soreness to touch, or in motion: *Bryon.*; — bloated: *Carb. veg.*

**Stomach**; illness and discomfort: *Phosphor.*; fullness in —: *Nux mosch.*; full feeling in —: *Baptis.*; from — to hip, sharp pain: *Bryon.*; — sensitive: *Phosphor.*, and burning: *Arsen.*; — sensitive to external pressure: *Arsen. Secal.*, to touch: *Mercur.*, even the bed-covering causes pain: *Laches. Sulphur*; — sensitive to pressure: *Arsen. Carb. veg. Colchic. Nux vom.*; — pains: *Opium.*; the least pressure is insupportable; excoriating pain in the epigastrium from touch and cough: *Bryon.*

**Epigastrium**; throbbing, pulsating: *Pulsat.*; painful sensibility: *Secal.*, of — to pressure: *Bryon. Colchic. Laches. Nux vom.*, to touch: *Mercur.*; tenderness and pain in —, remaining after touch: *Bryon. Nux vom.*; fullness in — with tension, and embarrassed respiration: *Mercur.*; distention with painful sensitiveness to touch: *Nux vom.*

**Liver**; region of — sensitive: *Phosphor.*, to touch: *Sulphur*; pain in —: *Mercur.*; painful sensibility: *Bellad.*; region of — puffed and engorged: *Mercur.*; in Hepatitis after Bryon., Laches., or Lycop.: ‖ *Mercur.*

**Spleen**; rumbling in region of — after Calcarea: *Lycop.*; — enlarged: *Cinchon. Coccul. Phosph. ac.*

*Rhus tox.*; — swollen, and painful to pressure: *Arsen.*; hardness of —: *Cinchon.*

**Abdomen**; heaviness in upper part: *Natr. c. Nux mosch.*; pain as if diarrhœa would set in: *Bryon. Cinchon. Veratr.*; sensation in — as if all were raw; distention immediately after drinking: *Nux vom.*; pinching, grasping in —: *Pulsat.*; colicky pains with looseness: ❙ *Veratr.*; frequent liquid stools: *Arsen.*; violent colic with gagging: *Nitr. sp. d.*; colic worse in the morning: *Bryon. Podolph. Sulphur*; burning in —: *Arsen.*; great soreness, and bloatedness of —: *Apis*; — very sensitive, and painful to touch: *Phosphor.*, and distention with hardness; rumbling in — as if there would be diarrhœa: *Veratr.*; sensitiveness in — aggravated after drinking water: *Mancin.*; incipient, or fully developed intestinal affection; indicated in the inflammatory stage, if diarrhœa has already set in, or Bryonia was not sufficient: *Rhus tox.*; ileo-cæcal region very painful to touch: *Arsen. Mercur. Phosph. ac.*, left iliac region: *Baptis.*; pain as from excoriation, or as if inflamed in the hypogastrium, especially when touched, with weakness: *Phosphor.*; intestinal ulceration, and hemorrhage: ❙ *Nitr. ac.*; sensation of heat in —: *Lachnath.*; surface of — hotter than the rest of the body: *Colchic.*; cæcal and peritoneal inflammation: *Bellad.*; the whole — compressed: *Rhus tox.*, drawn in: *Plumbum*, sunken in towards spine: *Bellad.*; hardness of —: *Arnic.*, with much flatulence: *Phosphor.*, and painful if pressed: *Opium*, *Secale*, and swelling as if from pent-up flatus: *Sulphur.*

**Soreness to touch**; in abdomen: *Apis. Chamon.*

*Hamam.* | *Laches.* || *Nitr. ac. Phosphor. Phosh. ac.*; — in precordia : *Mercur.* ; — in region of liver : *Arsen. Mercur. Rhus tox.* ; below the navel if pressed on : *Mercur. Phosphor.*

**Flatulence** ; fermentation in the bowels, with subsequent diarrhœa, and discharge of putrid flatus: *Carb. veg.* ; loud gurgling noise in the ileo-cæcal tract, dry tongue, heat, delirium on going to sleep : *Ginseng.* ; rumbling in the bowels, especially in the upper part : *Cinchon.* ; rolling and gurgling as if from much — : *Arsen.* || *Carb. veg.* | *Phosphor.*; rolling and grumbling : *Nux mosch. Sulphur.*

**Rumbling** : *Coccul.* | *Hyosc. Nux vom.* ; — during, and after drinking : *Phosphor.* ; — each time after drinking, or before every stool : *Mercur.* ; — before stool : | *Phosphor. Phos. ac.* ; — mostly in the evening in bed, or at night : *Bryon.* ; — as if diarrhœa would set in : *Phosphor. Veratr.* ; — before diarrhœa : *Laches.* ; — painful ; very offensive flatus: *Phosphor.* ; — with diarrhœa, and pinching and grasping pains : *Pulsat.* ; — with painless diarrhœa : *Phosphor.* ; — with diarrhœa of decomposed blood smelling horribly : *Carb. veg.*

**Tension** : *Cinchon. Colchic. Opium. Secal.*; — from accumulated gas, though much is discharged : *Phosphor.*; — with restless sleep, or heaviness in the upper part of abdomen : *Nux mosch.* ; very full and hard abdomen : *Phosphor.*

**Distention** ; with hardness : *Arnic. Bryon. Cinchon.* | *Coccul. Colchic. Laches. Opium. Sulphur. Veratr.*, and painful sensibility to touch : *Mercur.* : — as if from flatulence : *Carb. veg. Nux mosch.* ; —

with diarrhœa : *Cinchon.* ; — and rumbling : *Coccul.*; — before diarrhœa : *Laches.* ; — and constipation : *Lycop.*; — mostly in the afternoon : *Carb. veg.*; — with pain, as if bruised, and in the loins, when touched: *Phosphor.*; — with pain on pressure : *Hyosc.* ; — in the region of the navel, with severe pinchings, painful as if from pent-up gas ; very offensive flatus : *Rhus tox.*

**Bloated** : *Apis. Cinchon.*, not painful : *Helleb.*; swelling excessive : *Arsen.*

**Meteoristic distention** : *Arsen. Lycop.* ; with rumbling, gurgling, and painless, watery, grayish diarrhœa. : *Phosph. ac.* ; — with loud rumbling and gurgling in the intestines : *Carb. veg.*

Tympanitic distention : *Hyosc.*

**Tympanitis** : *Opium* ; — in the morning : *Cinchon* ; — and sensitiveness of abdomen, aggravated by drinking water : *Mancin* : — with pain in the back : *Colchic.*

**Eruptions** ; on abdomen roseola spots : *Arnic. Hyosc.* ; white miliary eruption : *Apis* ; petechiæ : *Bellad.*

Loins pain as if bruised : *Phosphor.* ; tearing: *Rhus tox.*

Groin sore, with involuntary diarrhœa : *Chamom.*

During the time of the crisis, when papescent stools, afford relief : *Arsen. Carb. veg. Mur. ac. Rhus tox.*

**Diarrhœa** ; occasional —: *Baptisia* ; watery —: *Arsenic. Colchic. Hyosc. Leptand. Mercur. Mur. ac, Opium* ; — more at night : Pulsat. ; — every half hour, each time after grumbling in the bowels,without pain : *Sulphur* ; — continues after the eruption : *Chlorum* ; painless —, abdomen bloated, hemorrhages, and slowly progressing reconvalescence : *Cinchon.* ; painless — with much rumbling noise : *Phosphor.* ; profuse —, stools streaked with blood, like flesh-water, and the tongue dry : *Phosphor.* ; colliquative —: *Secale* ; frequent and frothy, with tenesmus, even at night : *Sulphur* ; — excessive ; sour stools : *Calc. ostr.*

**Stools** ; whitish-gray: *Bellad. Phosph. ac.* ; — grayish : *Carb. veg.* ; — gray: *Phosphor.*

YELLOW, watery, and slimy: *Phosph. ac.* ; watery, or yellowish-brown, bloody, and cadaverous odor ; tongue brown, parched and hard: *Rhus tox.* ; yellow: *Bellad.*, watery and small: *Arsen.*, and slimy: *Cinchon. Phosph. ac.* ; liquid yellow, fetid, twelve to fifteen in twenty-four hours : *Nuphar* ; thin, yellowish, badly smelling with rumbling in bowels : *Phosph. ac.* ; sudden, thin, yellow, frothy, almost without fetor : *Rhus tox.*

GREENISH, watery, with mixed flocks: *Veratr.* ; greenish mucus : *Nitr. ac.* ; dark green and frothy: *Mercur.* ; green, slimy, acrid, with great tenesmus, sensitive abdomen : *Nitr. ac.* ; greenish-yellow : *Mercur.*

BROWNISH : *Carb. veg.*, soft : *Arsen.*, floating on the water : *Mercur.* ; brown, or white : *Arnic.* ; blackish-brown : *Veratr.*

BLACKISH : *Cinchon.* ; every hour : *Stramon.* ; black from decomposed blood : *Phosphor.* ; like coffee-dregs: *Arsen.*, only in the morning : *Podoph.* ; blackish, offensive stools with severe pain : *Colchic.*

BLOODY : *Apis. Arsen. Carb. veg.* ; dark : *Phosphor.* ; pitch-like, sticky : *Mercur.*

OFFENSIVE; fluid, with white flakes: *Colchic.*; like rotten cheese: *Bryon.* (*Hepar s. c.*); whether formed, or loose: *Laches.*; of decomposed blood, form and appearance of charred wheat straw, in larger or shorter flat pieces, together with portions more or less ground up, with hemorrhage and nose-bleed: *Laches.*; thin, and extremely offensive: *Laches. Opium. Psorin. Secal. Sulphur*; thin, very offensive with glairy slime, sometimes great urging: *Mercur*; fetid: *Baptis.*; dark, tarrying, mixed with bloody mucus; jaundiced condition: *Leptand.*; hemorrhage, with fetid stools, followed by great prostration: *Kreosot.*

PUTRID: *Carb. veg. Secal.*; black, burning, excoriating with restlessness and colic: *Arsen.*; colliquative: *Nux mosch.*; putrid, watery, with cutting and drawing pains in the abdomen and loins, extending to the thighs: *Nux vom.*; greenish, dark brown, odor like foul ulcers: *Arsen.*

GANGRENOUS, and bloody: *Phosphor.*

FOUL: *Apis. Arsen.*; like carrion: *Stramon*; cadaverous smell, involuntary, brownish, grayish, or bloody: *Carb. veg. Rhus tox.*

**Involuntary stools**; almost sudden, black-brown, every three hours: *Bryon.*, unnoticed: *Arnic. Arsen. Baptis. Bryon. Carb. veg. Cinchon. Colchic. Hyosc. Kali carb. Mur. ac. Opium. Phosphor. Phosph. ac. Psorin. Pulsat. Rhus tox. Secal. Sulphur. Zincum*; frequent — after meals, with rolling and rumbling in bowels: *Camphor*; — and urine: *Arnic. Bellad.* | *Mur. ac. Psorin.* | *Rhus tox. Sulphur*; — when flatus escapes: *Nux mosch.*, thin: *Veratr.*; — painful, foul, bloody: *Apis.*; — frequent: *Zincum*, and painful: *Apis*; — after constipation, or hard stool: *Nux mosch.*; offensive —: *Colchic.*, like carrion: *Carb. veg.*, — black-brown: *Byron.*; — thin, yellow: *Cinchon.*; — yellowish, mixed with phlegm: *Phosph. ac.*; — watery: *Colchic. Mur. ac. Nux mosch.*; — with white flakes: *Colchic.*, pieces of epithelium: *Zincum*; — at night in sleep: *Pulsat. Rhus tox.*; — with stupefaction: *Arnic.*

Paralysis of sphincter ani: *Hyosc.*

**Diarrhœa**; at night: *Rhus tox. Veratr.*; — with pressure in the abdomen, as if from gas: *Arnic.*; — after midnight: *Arsen.*; — early in the morning: *Sulphur*; — during cholera epidemics: *Veratr.*; — and bellyache, morning and evening: | *Kali carb.*; — with great epigastric oppression, black: *Dulcam. Mercur*; — with headache, and pain in all the limbs: *Rhus tox.*; — with bitter sour belching: *Baptis. Pulsat. Phosphor.*; — with empty belching: *Rhus tox.*; painful —, with great sinking of the forces: *Calc. ostr.*

Before the stool; pain in the abdomen: *Bryon. Laches. Rhus tox.*; rumbling: *Mercur. Phosph. ac.*; distention: *Arnic.*; weakness in abdomen, like fainting: *Veratr.*

With the stool; colic pains: *Arsen. Colchic.*; cutting; after sour things: *Ipecac.*; pinching pain, worse at night: *Pulsat.*; pain during, and after stool: *Veratr.*; painless stool: *Phosphor. Sulphur*; meteorism and loud rumbling: *Phosphor.*; tense abdomen: *Veratr. Verbasc.*; rumbling: *Arnic.*; tenesmus: *Nitr. ac. Sulphur.*

Frequent, small stools, excoriating the external parts: *Nux vom.*

Tearing pains in the limbs, during rest: *Rhus tox.*

Great weakness : *Arsen.* ; weakness, which compels to lie down, especially in the morning, or at night: *Bryon.* ; weakness, pale face, cold sweat on forehead: *Veratr.* ; feeble, stupid, insensible state, taciturn mood: *Phosph. ac.*

AFTER THE STOOL ; much exhausted, with griping in abdomen: *Mercur.* ; colic is gone: *Rhus tox.* ; great weakness: *Phosphor.* ; prostration : *Sulphur.*

He has six discharges like diarrhœa, then he faints ; first he had heat and warm sweat, then cold on the face and feet, with white tongue: *Sulphur* ; with chill: *Veratr.* ; if the diarrhœa does not cease when the rash comes out : *Calcar.*

**Flatus** ; putrid, offensive: *Arsen. Rhus tox. Sulphur* ; fetid : *Nux mosch.* ; copious escape of — with constant distention: *Carb. veg.* ; much —: *Sulphur* ; discharges of — without relief of tension : *Phosphor.*

**Blood** ; in streaks : *Phosphor.* ; bleeding from anus: *Arnic.*, bright, and does not clot : || *Nitr. ac.*, clotting and black : *Alum p. s.* ; hemorrhage from intestinal ulceration : *Arsen. Mur. ac. Nitr. ac. Phosph. ac.* ; fluid, bright red — from the rectum by the pound, fourteenth day, after a diarrhœa, and sensitiveness of regio iliaca dextra, seventh day, faintness with the slightest motion: *Nitr. ac.* ; reduced by hemorrhages to the verge of the grave : *Carb. veg.*; black —: *Mur. ac.* ; — of a tar-like consistency, in large quantities : *Hamam.* ; — and pus, meteorism, somnolency: *Carb. veg.*

Slimy discharges : *Cinchon. Mercur. Nitr. ac. Phosph. ac. Rhus tox.*

Bilious discharges: *Mercur.*; yellow or green discharges.

**Constipation**; alternating with looseness of bowels: *Arsen.*, or offensive watery diarrhœa: *Opium*; no diarrhœa: *Helleb.*; no stools for a long time: *Apis*; costive: *Baptis. Bryon. Hyosc. Lycop.*; "dog stools:" *Phosphor.*, six days: *Pulsat.*; — and vertigo, sour, or bitter eructations: *Nux vom.*

If constipation still exists, and hence the intestinal ulceration has not yet commenced, and if the sudamina and petechiæ have already broken out during the inflammatory stage: *Bryon.* If not costive, do not give Carb. veg. JAHR.*

**Bladder**; violent pain, and sensitiveness to the touch in region of —, passing of water, urine yellow, without sediment: ‖ *Lycop.*

**Urine**; frequent urination in small quantities: *Coccul.*; copious —: *Colchic. Mur. ac. Stramon.*; diminished —, and burning: *Arsen.*; difficult urination, with constant desire: *Secal.*; — dark: *Nux vom.*; saturated: *Bellad. Glonoin. Hyosc. Stramon.*; — scanty; small quantities of dark brown —: *Pulsat. Sulphur. Veratr.*; pains when urinating: *Canthar.*; pains in bladder, — has red sediment: | *Lycop.*; suppressed —: *Canthar. Colchic. Secal. Stramon.*, or retained: *Arsen.* ‖ *Hyosc. Stramon.*, as if from a closing of the bladder, or loss of its power: *Opium*; — passes involuntarily,

---

* I have not yet lost a single patient, in whom, up to the time of the crisis, when the papescent stools afford relief, the bowels remained costive. JAHR.

I said the same more than twenty years ago, and can repeat it now; hardly in the *third week* the non-appearance of a stool is to be regarded. *The same applies in child-bed.* C. HG.

is turbid, like horse urine: *Apis*; unconscious flow of —: *Apis*; involuntary: *Arsen. Colchic. Helleb. Hyosc. Lycop. Opium. Phosphor.*; — is passed unconsciously in bed: *Mercur.*; involuntary all night: *Arnic. Arsen. Bellad. Hyosc. Rhus tox*; — copious, involuntary: *Stramon. Veratr.*; paralysis of sphincter vesicæ and ani: *Hyosc.*; involuntary stool and —: *Bellad.*

WHILE URINATING; stool escapes: *Mur. ac.*

WATERY: ||*Mur. ac.*, clear, transparent and acid reaction: *Mur. ac. Phosph. ac.*; alkaline and offensive: *Baptis.*; clear, very high colored: *Nux mosch.*

DARK RED; without sediment, brownish-red, brown with burning: *Pulsat.*

BROWN: *Bryon. Pulsat. Arsen. Sulphur. Veratr.*

DARK: *Carb. veg. Mercur. Nux vom.*

First clear, then white as if mixed with chalk: *Mercur.*

**Turbid**: *Carb. veg. Arsen.*; — like horse urine: *Apis*; — after standing: *Sulphur*; — and white sediment: *Phosphor.*; — and reddish; dark colored: *Carb. veg.*; greenish, or dark brown, when passed, and does not become clear on standing: *Arsen.*; after depositing a sediment, not clear: |*Phosphor.*

**Urine**; more albumen in —: *Rhus tox.*; — coagulates, like milk: |*Phosph. ac.*; white sediment in —: *Mercur. Phosphor.*; — colors linen slightly red: *Bellad.*; — leaves, if voided in bed, a red, sandy stain, like brick dust: *Lycop.*: — leaves a whitish sediment on the parts, legs and sheets: *Mercur.*; — has a gray sediment: *Pulsat.*; — first clear, then white as if mixed with chalk: *Mercur.*

**Smell**; like violet roots: *Phosphor.*; strong —:

*Carb. veg.*, like horse urine: *Apis*, sharp, disgusting: *Phosphor.*, very offensive: *Baptis. Sulphur*; strong ammoniacal —, turbid, deposits white sediment: *Phosphor.*; fetid —: *Baptis.*; intolerable stench, and involuntary: **|** *Arsen.*

**Genitals**; great excitement, nymphomania threatens: **|** *Phosphor.*; bleeding from — with no relief: *Rhus tox.*; threatened miscarriage: *Sabin. Secal.*

**Speech**; nasal, indistinct: *Laches.*; the few words he says, he whispers; but not irrelevant: *Nitr. sp. d.*; — unintelligible, lisping, stammering, as though the tongue were too heavy: *Arsen.*; voice light and weak: *Veratr.*; — weak and trembling, or hoarse, coarse, or crowing: *Arsen.*; cries until the — is lost, or he becomes hoarse: *Stramon.*; voice hoarse and hollow: *Secale. Spongia*; laryngotyphus: *Kali hydr.*; catarrhal affection of larynx, with rough voice: *Hepar s. c.*

**Sighing**: *Bryon. Opium. Secal.*; inclined to sigh; expands whole chest in —, with chill: *Ipecac.*

**Breathing**; audible and accelerated: *Colchic.*; — more frequent: *Mur. ac.*; — rapid and labored: *Mercur.*; — interrupted: *Phosphor.*; intermittent, with moaning: *Opium*; — irregular, and intermittent: *Colchic.*; — slow: *Helleb.*, deep drawn, and sighing: *Opium*; sighing, groaning, and moaning, and a peculiar, sour smell of the body, with, or without sweat: *Bryon.*; — short and anxious, oppressed, rattling: *Arsen.*; — short, with anxiety, and vertigo: *Phosphor.*; — short, cough, cold hands and feet, wants fire in the room: *Carb. veg.*; — short after each cough: *Phosphor.*; rattling —: *Hyosc.*, and moaning sounds in chest, and loud sounds through the nose:

*Cinchon.*, and loud —: *Opium*, and hard —: *Phosphor.*, and cough: *Nitr. ac.*; — deep: *Nux mosch. Opium*; mucous râle during stupor, or furious delirium: *Hyosc.*; the more snoring, the darker the face becomes: *Opium*; stertorous —: *Laches. Opium*; — loose, rattling, loud and difficult: *Opium*; gray phlegm comes up: *Nitr. sp. d.*; cold —: *Carb. veg.*; breath is hot to back of hand: *Phosphor.*; offensive, putrid breath: *Arnic.*; very offensive breath: *Baptis.*; oppression of chest, and difficulty of —, with pleuritic stitches in chest, and harassing cough, with thick yellowish, or reddish sputa: *Phosphor.*; anxious respiration with strong heaving of the chest: *Opium*; want of breath, anguish in chest as if pressed together: *Phosphor.*; embarrassed — from tension in stomach: *Mercur.*; dyspnœa: *Laches.*; difficult, anxious — with sighing and hiccough: *Secal.*; oppressed — with extreme prostration: *Arsen.*; gasping — could not draw a full breath: *Baptis.*; sudden spells of suffocation, mostly in evening, lying down, with or without cough: | *Arsen.* | *Phosphor. Pulsat.*

Impending paralysis of lungs: *Nitr. ac.*; cough ceases, the collected mucus cannot be expectorated: *Moschus*; rattling phlegm in wind-pipe: *Arsen. Phosphor.*; cough ceases, the collecting secretions cause loud rattling breathing: *Carb. veg.*

**Cough**; suddenly changes from a loose one to a dry and hoarse one, with anxiousness and dyspnœa: *Spongia*; — hard, with stitching pain in chest: *Bryon.*; a dry — and difficult breathing: *Laches.*; dry, short, hacking —, evening in bed, as if from roughness, or dryness in the larynx: *Bryon.*; harassing — all night, no expectoration in the day-time: *Rhus tox.*; dry —: *Arsen.*, and sore throat from bronchial irritation: *Bellad.*; — with very little expectoration, makes them worse: *Rhus tox.*; hard —, with tightness in the chest: *Phosphor.*; — with pain in the head as if it would burst: *Phosphor.*; troublesome — which causes pain in the forepart of the chest, and wakes from sleep: *Phosphor.*; severe —, with pressing headache the whole day: *Phosphor.*

**Phlegm**; mucus in throat, neither able to swallow, nor to expectorate: *Baptis.*; rattling of — on chest: *Carb. veg.*; moist râles in bronchial tubes, audible at a distance, from infiltration of lungs: *Ant. tart.*; loose rattling cough and breathing: *Nitr. ac.*; in typhus with children: *Bellad. Bryon.*; loose cough: *Lycop. Phosphor.*, without expectoration, with pain and sense of excoriation in the chest, so that he fears to cough: *Phosphor.*; tenacious mucus from throat, troublesome: *Apis.*

**Sputa**; tough, like gelatine: *Bryon.*; — first transparent, then yellow-colored, with or without impeded breathing: *Ant. tart.*; — transparent, or thick, yellowish, or reddish: *Phosphor.*; mucus yellow, red, and transparent, hangs on the tongue: *Phosphor.*; — viscous, hangs on the lips: *Phosphor.*; — hard to expectorate, with cough, in reconvalescence: **|** *Arsen.* **|** *Seneg.*; catarrhal and pulmonary difficulties in the inflammatory stage: *Bryon. Phosphor.*; — copious in the morning: *Bryon.*; — streaked with blood: **|** *Laches.*; frothy, bloody mucus with violent, almost constant cough: *Kali carb.*; — brownish, bloody, with irregular pulse: *Nitr. ac.*; — bloody, with severe cough : |*Rhus tox.* ; — covered with blood : *Rhus tox.* ; — slimy, bloody : *Laches.* ; bloody froth with the —, cough excites bilious vomiting : *Carb. veg.*

**Cough** ; in the evening in bed : *Bryon.* ; worse from evening till midnight : *Phosphor.*

**With cough** ; painful shocks in the head : *Calc. ostr.* ; pressing headache all day : *Phosphor.* ; bursting pain in head : *Phosphor.* ; sore throat : *Bellad.* ; pain in epigastrium : *Bryon.* ; stitching pain in liver : *Bryon.* ; roughness and dryness in larynx : *Bryon.* ; dry cough, with difficult breathing : *Laches.* ; after each cough, short breath : *Phosphor.* ; tightness in chest : *Phosphor.* ; pain in forepart of chest : *Phosphor.* ; pleuritic stitches in side of chest ; restless and anxious : *Bryon.* ; wakes from sleep : *Phosphor.*

Fever is increased by the cough : *Calc. ostr.*

**Chest** ; pressure on the lower part on — : *Phosphor.* ; stitches in the left side : |*Acon.* ||*Bryon.*, in lungs, or pleura, with a dry cough : *Bryon.* ; pleuritic stitches when inhaling : *Arnic.*, when coughing : *Bryon.* ; shooting pains in left side, worse in the evening, and on motion : *Bryon.* ; constant tickling under the middle of the sternum, causing a hacking cough, worse from talking, or moving : *Calc. ostr.* ; aggravation of thoracic pains by the least motion, or from breathing : *Spigel.* ; inflammatory affection of lungs, with rattling cough and breathing : *Nitr. ac.* ; effused fluid in pleura after pleuritis : *Sulphur* ; inflammation of lungs, or catarrh, especially during the commencement of infiltration, recognizable by the crepitation sound : ||*Sulphur* ; infiltration of lower lobes : *Rhus tox.* ; hepatization : ||*Phosphor.*

Bronchitis: *Bryon. Mercur. Rhus tox.*; whole process falls on the lungs: *Phosphor.*, if it does not succeed: *Bryon. Tart. emet.* or *Nitr. ac.* (JAHR.)

Pneumonia: *Phosphor.*; tuberculosis of lungs: *Kali carb.*

Pneumonic symptoms throw all the others into the shade; next to *Bryon.* and *Rhus tox.*: **|** *Phosphor.*; if the patient is tormented by a violent, racking cough, either dry, or with thick, yellow, tenacious sputa, spits as in pneumonia: *Laches.*; lungs filled with blood: **|** *Nitr. ac.*

In *pneumo-typhus: Apis. Bellad. Bryon. Mercur. Phosphor. Pulsat. Rhus tox. Sulphur*, with great rattling, dyspnœa, œdema pulmonis: *Ant. tart.*

Œdema of lungs threatens during the mucous râle: *Ant. tart. Carb. veg. Phosphor.*

Paralysis of lungs threatened: *Ant. tart. Nitr. ac.*

**Heart**; pressure and anxiety: *Rhus tox.*; — palpitates: *Ignat.*, during the aggravations, preceding the miliary rash, fourteenth day; tremulous pulse, anxiety, restlessness, redness of the face, delirium, jerkings, especially in children; short hacking cough; excessive diarrhœa: *Calc. ostr.*; excited beating of —; anxiety, with tossing in bed; says but little, only complains of anxiety; says nothing from weakness of body and mind; excess of sensibility to sounds, to talking and to light; great indifference to all things, even to life; dull and weak in the head: *Arsen.*

Paralysis beginning; face and limbs grow cold, become covered with cold sweat; a picture of complete torpor of all vital functions (thus differing entirely from that of Arsen., which is always associated with erethism): *Carb. veg.*

Slow action of heart: *Helleb.*; frequent and small beats: *Coccul.*

Circulation without energy; blood stagnates in the capillaries, causing cyanotic blueness of face, lips, and tongue: *Carb. veg.*

**Pulse**; very low: *Helleb.*; — weak, feeble: *Apis. Arsen. Carb. veg. Hyosc. Laches. Mercur. Phosphor. Phosph. ac. Psorin. Pulsat. Veratr.*, and intermitting: *Apis. Veratr.*, and quick: *Arsen.*

WEAK and irregular: *Hyosc.*; — and frequent (sixth week), moving of limbs causes drawing pains along the spine: *Ferr. carb.*; — and very frequent, irritated, without energy, 120 to 130: *Mur. ac.*; — and rapid: *Mercur.*

SMALL: *Arsen. Carb. veg. Colchic. Hyosc. Laches. Phosphor. Phosp. ac. Psorin. Pulsat. Rhus tox. Secale. Sulphur. Zincum*; — and frequent: *Colchic.*: —, weak and trembling: *Arsen.*; —, weak and intermitting: *Arsen. Zincum*; —, weak and scarcely perceptible: *Zincum*; —, weak and quick: *Phosphor. Rhus tox.*; —, weak and contracted: *Colchic. Secal.*; —, weak, slow and intermitting: *Secal.*; — and irregular: *Arsen.*; — and thread-like: *Hyosc.*; — and hardly perceptible: *Hyosc. Zincum.*

WEAK and small: *Arsen. Carb. veg.* | *Hyosc. Laches.* | *Phosphor. Phosph. ac. Psorin. Pulsat. Rhus tox.*: —, small and frequent: *Laches.*, and intermitting: *Phosph. ac.*, scarcely perceptible: *Carb. veg.*; —, small and quick (130): *Psorin. Sulphur*, almost suppressed: *Pulsat.*, and rapid: *Arsen.*, and trembling: *Arsen.*, and miserable: *Nuphar.*

6

THREAD-LIKE, stringy, wiry: *Arsen.* ‖ *Carb. veg. Colchic. Hyosc.* ‖ *Rhus tox.*, and variable: *Baptis.*, and quick: *Colchic. Phosphor.*

SCARCELY PERCEPTIBLE: *Carb. veg. Hyosc. Zincum*, and quick: *Colchic.*, and small: *Hyosc.*, and frequent: *Zincum*; very frequent: *Camphor.*

PULSELESS: *Arsen. Carb veg. Colchic. Jodium*, and unconscious: *Mercur.*

FREQUENT: *Arsen. Bellad. Carb. veg. Colchic. Glonoin. Hyosc. Jodium. Laches. Stramon. Zincum*; —and empty: *Arsen.*; — large and hard: *Jodium*; — and small: *Arsen. Coccul. Colchic. Zincum*; irritated, but without energy: *Mur. ac.*; —, small and trembling: *Arsen.*, and intermitting: *Zincum*; —, small and weak: *Laches.*, and sunken: *Arnic.*, and intermitting: *Phosph. ac.*, scarcely perceptible: *Zincum.*

ACCELERATED and full: *Phosphor.*; —, over 100: *Arsen. Baptis. Phosph. ac.*, 110 to 130, without energy: *Mur. ac.*, 120 to 140 in the minute: *Bellad. Kali carb. Laches. Pulsat. Sulphur.*

QUICK: *Arsen. Colchic. Psorin. Pulsat. Rhus tox. Sulphur. Zincum*; —, weak and intermitting: *Arsen.*; — and small: *Phosphor. Rhus tox.*; —, weak and small: *Psorin. Pulsat. Sulphur*; — and thread-like: *Colchic.*, and trembling: *Arsen. Phosphor.*

RAPID: *Hyosc. Mercur.*, and intermitting: *Hyosc.*, and weak: *Mercur.*; —, weak and small: *Arsen.*

INTERMITTING: *Apis. Arsen. Hyosc.* ‖ *Kali carb. Mur. ac. Phosph. ac. Secale. Veratr. Zincum*; — and weak: *Apis. Arsen. Veratr.*; — small and frequent: *Zincum*, and weak: *Phosph. ac.*; omitting every third beat: *Mur. ac.*

SLOW: *Opium. Secal.*; only 80: *Helleb.*, and full: *Baptis.*

CONTRACTED: *Colchic. Secal.*

HARD, large and frequent: *Jodium*; — and full, 98: *Baptis.*; carotids beat violently: *Bellad. Glonoin. Hyosc. Stramon.*

FULL: *Opium. Phosphor.*, and accelerated: *Phosphor.*; 120: *Gelsem.*; — and quick: *Baptis.*, and slow: *Baptis. Opium*, and hard: *Baptis.*

EMPTY: *Arsen.* See Imperceptible.

CHANGING frequently: *Apis. Baptis.*

INCREASING by least motion: *Jodium.*

**Outer Chest**; eruption of a pinkish-blue red color, extending over whole left side: *Stramon.*; roseola spots: *Arsen. Hyosc.*; petechiæ: *Bellad.*

RASH; red: *Stramon.*, white: ❙ *Valer.*; miliary eruption from neck to chest, then all over: *Phosph. ac.*

Throbbing in the arteries of the throat: *Phosphor.*

**Neck**; stiff: *Bryon.*, with pain in the bones, as after taking cold in a copious sweat, with pressing to forehead: *Nux mosch.*

**Back**; spine sensitive when fever is coming on: ❙ *Nux vom.*; pain in —: *Colchic.*; rending along spine from moving either hands or feet: ❙ *Ferrum*; loins and legs, as if bruised, and weak; after the slightest exertion, weakness with inclination to lie down: *Nux mosch.*

Great heat of the trunk, with cold sweat on head and limbs: *Phosphor.*

Red rash: *Stramon.*; miliary rash beginning on the neck: *Phosph. ac.*; petechiæ on neck: *Bellad.*

### LIMBS.

**Upper limbs**; arm gets livid, and goes to sleep: *Kali carb.*

Hands; trembling automatic motion of —, with coldness of the limbs: *Zincum*; — tremble violently when trying to lift, or move them: *Gelsem.*; — weak: *Arsen.*; one — hot, the other cold: *Pulsat.*; cold sweat on —: *Carb. veg.*, on — and upper limbs: *Zincum.*

Fingers cold after drinking: *Tarax.*

**Lower limbs**; thighs, weariness: *Phosphor.*; — weary, even while rising from long continued sitting: *Pulsat.*; — feel weak: *Arsen.*, especially in recovery, they think they will have paralysis: | *Selen.*; — tremble when trying to walk: *Gelsem.*; tearing in —, most unbearable when at rest, relieved by moving them (Rhus tox. did no good): *Tarax.*; — weak, evening or morning, sluggish, disposed to sweat, and trembles: *Carb. veg.*; attacks of jerking in —: *Coccul.*; aching, and sense of deadness in —: *Bryon.*; heaviness in — in the morning, with nausea, sinking and drowsiness: *Mercur.*

Knees; weak: *Arsen.*, while sitting, or in quick motion: *Phosphor.*

Feet; great heaviness of —: *Coccul.*; weak —: *Arsen.*; cold —: | *Lycop.* | *Zincum*, all night: *Jodium*; cold sweat on —: *Carb. veg.*

Toes; sore between —, and sweaty: *Baryt. Silic.*

**All the limbs**; muscles refuse to obey the will: *Gelsem.*; — trembling, or jerking: *Coccul.*

Vanishing of power, like fainting, near losing his consciousness: *Sulphur*; numbness: *Secal.*; sluggish, more in the forenoon, with heaviness: *Phosphor.*; weak: *Secal. Veratr.*; inclination to lie down: *Veratr.*; heaviness: *Opium*, and weariness: *Bellad.*, with numbnesss: *Secal.*, especially of thighs: *Cinchon.*, and of the whole body, with chilliness: *Pulsat.*, as if from extreme fatigue: *Arnic.*, and peculiar lassitude, with headache, white coated tongue, loss of appetite, restless sleep: *Bryon.*; and weak from morning to evening: *Sulphur*, and weariness, especially in the legs, on rising from sitting: *Bryon.*

Great weariness; weakness in the evening, with depression of spirits: *Mercur.*

Painfulness extending from the elbows and knees: *Laches.*; severe pain in the limbs: *Rhus tox.*; severe rheumatic pains, worse in rest, somewhat ameliorated by moving and changing position: *Rhus tox.*; bruised feeling: *Bryon. Rhus tox.*; coldness of the limbs: *Zincum*, hands and feet: *Arsen. Colchic. Lycop.*; one hand and foot hot, the other side cold and red, evening and morning: *Pulsat.*; one foot hot, and the other cold: *Lycop.*; limbs cold, covered with cold sweat: *Carb. veg.*

**Motion**; desire for frequent and constant movement, which gives temporary relief to the patient, pain better in motion: *Rhus tox.*; fidgety feet: *Zincum*; averse to effort: *Sulphur*, to all efforts of body and mind: *Cinchon.*, and dullness: *Arsen.*, wants to lie quiet: *Bryon.*; on rising from lying, vertigo: *Arnica. Nux vom.*; on raising head, vertigo: *Cinchon.* (see Head); on rising from sitting, loss of strength; the same at the beginning of walking; weary legs: *Bryon.*; on moving, soreness in stomach: *Bryon.*; on moving quickly, weakness of knees: *Phosphor.*

WALKING; inordinate weariness from a short walk; can walk but a few minutes on account of weakness: *Pulsat.*; even the least walk produces great fatigue and headache: *Phosphor.*

Pain in all the limbs: *Bryon.*; aggravated darting-tearing pains: *Byron.*

General aggravation of pains from motion: *Bellad. Byron.*

In the suffering parts shooting, or jerking, tearing pains; in the head, throat, chest, abdomen, etc.: *Byron.*

From the least labor weakness, and exhaustion with heat, rush of blood and trembling: *Mercur.*; on the least exertion, loss of strength: *Bryon.*; after the slightest movement, immediately great weariness: *Nux vom.*, great weakness: *Mercur.*; exhaustion: *Laches.*, he must sit down: *Coccul.*, fainting: *Coccul. Nitr. ac.*, chilliness (in the early stage): *Nux vom.*, in the morning: *Calc. carb.*

Overestimates his strength, ventures to get up, and then sinks down on to the floor: *Apis. Arsen. Lycop. Natr. mur.*; can only walk a few steps without support: *Stramon.*; unable to walk, sinks down after a few steps: *Arsen.*

Every motion during the fever makes the patient feel sick: *Fluor ac.*, causes sweating: *Cinchon.*

AFTER OVER-EXERTION; of the body: *Rhus tox.*; of body and mind: *Cuprum.*

**Restlessness**; anxious to go from one bed to another: *Arsen. Bellad. Calc. ostr. Chamon. Cina. Hyosc. Mercur. Sepia. Rhus tox. Veratr.*; turns from one place to another: *Arsen. Hyosc. Rhus tox.*; lying on back, suddenly sits up, then lies down again: *Hyosc.*; restless, tossing about: *Apis. Bellad. Canthar. Rhus tox.*; continually changing position: *Arsen. Bryon. Cinchon. Rhus tox.*; she has no rest day or night: *Sulphur*; great —: *Hyosc. Rhus tox.*, and anxiety, manifesting itself in constantly moving head and limbs, whilst the trunk lies still, on account of too great weakness: *Arsen.*

General restlessness; in the muscles, with vertigo: *Nux mosch.*, with dilated pupils: *Nux vom.*; all night, puts out hands: *Phosphor.*; in the evening, tosses hands about: *Phosph. ac.*; constant inclination to stretch limbs or change their position: *Cinchon.*; can't lie long anywhere: *Baptis.*; wants to rise up: *Pulsat.*; great nervous restlessness: *Baptis.*, of body and limbs: *Camphor*; every position feels too hard: *Arnic.*; feels better when lying down: *Bryon.*; rest relieves colic: *Arsen.*; weakness of the whole body, which compels lying down: *Arnic. Mercur. Pulsat.*; giddiness compels lying down: *Phosph. ac.*

**Standing**; he can hardly stand erect on account of great weakness; great weariness at nine o'clock, A. M., with heaviness in all the limbs and almost an unconquerable inclination to sleep: *Coccul.*; he cannot stand up, but only lie or sit; if he stands, he has the greatest anxiety with nausea and cold sweat on the forehead: *Veratr. alb.*

After stooping, cannot rise: *Rhus tox.*

**Sitting**; on sitting up he has nausea: *Pulsat.*; —, knees weak: *Phosphor.*; stupid, as if drunk, on rising dizzy, as if he would fall forwards or backwards: *Rhus tox.*; —, giddiness: *Phosph.* ac.

**Lying**; wants to lie down: *Coccul.*, in one spot (in the beginning): *Rhus tox.*; strong desire to lie down, and considerable relief on doing so: *Nux vom.*; sluggish, with constant desire to lie down or to sit: *Nux vom. Pulsat.*; must lie down, sitting does not suffice: *Pulsat.*; strong inclination to lie down: *Stramon.*; painful to lie on the left side: *Stramon.*

Shooting pains, worse during rest: *Rhus tox.*

During rest intolerable tearing pains, only in the lower limbs: *Tarax.*; in all the limbs: *Rhus tox.*

**Positions**; lies quiet, without any complaint: *Arnic.*; lying with head thrown back: *Baptis.*; lying on back: *Arsen. Laches. Phosphor. Zincum*; limbs drawn up: *Helleb. Pulsat.*; knees drawn up: *Arsen. Pulsat.*; legs bent to belly, forearm stiffly bent on arms: *Bellad.*; eyes half open, abdomen sunk in: *Colchic.*

Sliding down in bed: *Apis. Baptis. Helleb. Zincum*; slips down to the bottom of the bed: *Arsen.*, into one heap with moaning: **||** *Mur. ac.*

Debility quickly following the erethic stage: *Rhus tox.*

WEARINESS: *Cinchon. Coccul.*; — of limbs: *Stramon.*; — all over, feel as if they had overdone themselves: *Arsen.*; — in the whole body: *Pulsat.*; — most in thighs: *Phosphor.*; painful — in the limbs, as if after a long walk: *Cinchon.*; — as if after exertion: *Colchic.*; unconquerable —, like intoxication: *Opium*; — as if after sickness: *Sulphur*; always fatigued and weak: *Sulphur.*

WEAKNESS: *Camphor. Coccul. Opium. Rhus tox. Secal. Stramon.*; — suddenly in the midst of the disease: *Chlorum. Nitr. sp. d.*; frequent sudden attacks of —: *Phosphor.*; — sometimes more in the upper, sometimes in the lower limbs: *Secal.*; — intolerable, with giving way of the knees: *Mercur.*; in attempting to move, too weak to control his movements: *Gelsem.*; — of limbs, with trembling after every effort: *Sulphur*; —, ready to fall; with heat, rush of blood and trembling: *Mercur.*; on getting up, sinks down to the floor: *Arsen. Apis. Natr. mur.*; the greatest —, especially in the legs, knees, feet, and hands, which tremble, with inability to walk more than a few steps without sinking down: *Arsen.*; universal — in the morning, as if after too little sleep: *Veratr.*; — and anxiousness, with staggering: *Arsen.*; with relaxation of body and mind, with insensibility: *Cinchon.*; — with aversion to all external objects, persons, and things, with drowsiness: *Opium*; — ready to fall, with inexpressible sense of illness in body and mind, which compels him to lie down: | *Mercur.*; — and exhaustion with heat, rush of blood, trembling from the least labor: *Mercur.*; — with strong disposition to sweat during movements and in sleep: *Cinchon.*; — and sweat after fever: *Psorin.*; — with inclination to sleep: *Laches.*; — weariness and bruised soreness, which compels to lie down, and yet every position feels too hard: *Arnic.*; — with sensibility of the surface: *Colchic.*; — and great lassitude: *Bryon.*; — with the vertigo: *Secale.*

LASSITUDE; the whole day: *Sulphur*, the whole body sluggish: *Arnic.*, general relaxation with great nervous weakness: *Phosphor.*

Debility: *Carb. veg. Phosph. ac.*; — as if after severe sickness: *Natr. mur.*; — and nervous prostration: *Baptis.*; greatest debility: *Nitr. sp. d.*; with sliding down in bed: *Mur. ac.*; with trembling of the whole body, and coldness of the limbs: *Hyosc.*; — and weakness with sleepiness in the afternoon: *Bellad.*

**Prostration**: *Chlorum. Colchic. Gelsem. Mercur. Rhus tox. Stramon. Veratr.*; very great —: *Arnic.*; uttermost and overwhelming —: *Arsen.*; lying in a state of stupid apathy, senses inactive, very taciturn, and insensible to every external impression, abdomen much distended, many gastric ailments, and stools involuntary: *Phosph. ac.*; — with inability to leave the bed; falling of the lower jaw and eyelids: *Arsen.*; exhaustion: *Rhus tox.*; —, averse to every effort, even to speak: *Sulphur*; — and anorexia: *Rhus tox.*; — and emaciation; *Mercur.*; — rapid: *Arsen.*

Sense of internal illness as of impending disease: *Cinchon.*; attacks as if body and mind were unstrung: *Mercur.*

Sinking of Strength: *Arnic. Hyosc.*; loss of all strength: *Phosph. ac.*; rapidly: *Phosphor.*

Sinking of all the Forces: *Cinchon. Rhus tox.*; general and rapid: *Arsen. Phosphor. Secal.*; sudden: *Colchic. Nux vom. Veratr.*; in ten hours he can hardly speak or walk: *Colchic.*; with muttering delirium, apathy, loss of sense, bluish redness of the face, and a feeble, intermittent pulse: *Veratr. alb.*; paralytic, even in sitting, but mostly when moving: *Nux vom. Opium*; with disposition to sleep: *Veratr.*; rapid sinking, dry tongue and skin, after bloody stools: *Arsen.*; withered bluish skin: *Carb. veg.*; sinking down exhausted: *Veratr.*

Inexpressible sense of illness in body and mind: *Mercur.*

FAINTING: *Secale.*; sudden —: *Bryon. Camphor. Coccul.* | *Tarax.*; — and vertigo with every attempt to leave the bed: *Opium*; — from bodily movement, with spasmodic distortion of the facial muscles: *Coccul.*; sense of illness and discomfort in the whole body, especially in the stomach, even in the open air: *Phosphor.*; faintness and fever and trembling: *Laches.*

Heaviness and dullness of body and mind, loathsome sensation of the whole body, with weakness of the joints, especially of the knees, while sitting and in quick motion: *Phosphor.*

Weakness and weariness develop themselves mostly as heaviness: *Pulsat.*

Lies speechless with open eyes and stiff limbs: *Opium.*

Appears to feel almost nothing, to be almost immovable and yet not quite paralyzed: *Nitr. sp. d.*

All strength gone, settles down in the bed, paralysis threatens: *Arsen. Carb. veg. Mur. ac. Rhus tox. Nitr. ac. Moschus*; nearly at the verge of the grave: *Nux mosch.* 2^c^.

Debility as if the parts would be paralyzed: *Rhus tox.*

Paralysis of one or more parts: *Caustic.*

Protracted reconvalescence: *Psorin.*

If the strength alone is still wanting: *Veratr.*

Anxiety in the body with disposition to tremble: *Nux mosch.*

Nervous tingling, lameness: *Acon.*

Cow walk (gressus vaccinus): | *Laches.*

Staggering gait, with difficulty in walking direct to a given point, and anxious weakness: *Arsen.*

TREMBLING: *Arsen. Bellad. Hyosc. Ignat. Lycop.*; — of limbs: *Arsen.*, and internal — with fever and faintness, evenings: *Laches.*; — weakness: *Pulsat.*; — of the body with prostration: *Carb. veg.*; — with sense of weariness in the limbs (in the early stage): *Bellad.*; — of the whole body, or one or more of the limbs: *Stramon.*; — after every effort: *Sulphur*; — of the part moved, in every effort, even of the protruded tongue: *Bellad. Laches. Secale.*; — and jerking of the limbs: *Apis*; — in attacks, and jerking of eyelids, muscles of face and limbs, fits of fainting from bodily movement, with spasmodic distortion of the facial muscles: *Coccul.*; — in the morning with jerking of the limbs: *Phosphor.*

**Motions**; automatic — of muscles: *Camphor. Rhus tox.*, of hands and feet: *Coccul. Rhus tox.*; involuntary —: *Bellad. Colchic. Hyosc. Rhus tox. Stramon.*; constant odd — of limbs and body: *Stramon.*; sudden startings: *Canthar.*; twitchings, the fourteenth day: *Colchic.*; frequently raises or jerks the head from the pillow: *Stramon.*

JERKING and turning of hands: *Opium*; violent — of the limbs, shaking whole body, awake or asleep: *Lycop.*; — of the limbs and tendons; debility and weariness of the whole body: *Hyosc.*; — of the tendons: *Ignat.*, with pale face and cold hands, and small, hard, rapid pulse: *Bellad.*

SUBSULTUS TENDINUM: *Chlorum. Lycop. Psorin. Rhus tox. Secale. Sulphur. Zincum*; violent: *Hyosc.*

Clonic spasms: *Canthar.*

Spasmodic motions, convulsions: *Hyosc. Ignat.*; convulsive motions of mouth or face: *Bellad.*; intermittent convulsions every two or three hours, recur at the same hour: *Moschus.*

Clonic spasms of upper and lower limbs with paralytic weakness: *Lauroc.*; clonic spasms, from spinal marrow: *Stramon.*

No loss of consciousness with spasms: *Lauroc.*

### SLEEP.

**Sleeplessness**; with desire to sleep: | *Bellad.*; extreme prostration and sleepiness, but unable to sleep: *Mercur.*; impossibility to sleep, though feeling very sleepy: *Opium*; great desire to sleep, but cannot: *Mur. ac.*

**Sleepiness**; unconquerable inclination to sleep: *Coccul. Hyosc.*; with weakness: *Cinchon.*; he cannot prevent sleep when seated at work in the daytime: *Sulphur*; while answering a question falls into a deep sleep before finishing: *Arnic. Baptis. Hyosc.*; — like coma: *Phosphor. Pulsat. Secale*, and stupid: *Gelsem.*; — in afternoon: *Bellad.*; sudden — in the evening, cannot rouse herself from it: *Rhus tox.*; — through the whole day: *Bryon.*; very weak and sleepy the whole day: *Sulphur*; often through the day compelled to sleep whole hours: *Pulsat.*; he must sleep all the time: *Bryon.*; he sleeps on his chair, in a half-conscious state: *Veratr.*; falls asleep while sitting: *Mercur.*; if left alone, he sinks into slumber: *Helleb.*; — the whole day, with anxiety, restlessness, sadness, dry lips, and a constant desire to lie down: *Rhus tox.*

GREAT DROWSINESS: *Bellad.*; slumbering all day: *Phosph. ac.*

Slumber with frightful dreams, changing into delirium: *Bellad.*

Frightful or imaginary visions on closing the eyes: *Arsen. Bellad. Calc. ostr. Sambuc.*; on going to sleep delirium: *Ginseng.*

Uncommon sleepiness as if from stupefaction of the head: *Nux vom.*

Great sleepiness with giddiness, walks with the eyes shut: *Nux mosch.*

Sleepiness with weakness in all the limbs: *Laches.*

Easily roused out of sleep and soon is conscious: *Phosph. ac.*

Gentle sleep: *Hyosc.*

Constant slumbering: *Phosph. ac.*

Too great disposition to sleep, which is too profound; slept twelve hours and would longer if not wakened: *Mercur.*

Cannot go to sleep because she cannot get herself together; head feels scattered about, tosses about to get the pieces together: *Baptis.*

Uninterrupted sleep for three days: *Veratr.*; like in a deep sleep: *Mercur.*; deep sleep after delirium: *Secale.*

He cannot be roused or only with great difficulty: *Opium*; continued profound sleep: *Veratr.*

Sleep with loose rattling respiration: *Opium*; — with stertorous breathing: *Opium. Stramon.*

Stupefying slumber the whole day: *Secale*, also at night, with increased thirst: *Opium*; deep and long-continued sleep: *Secale.*

In stupefying sleep, stertor, especially during expiration: *Opium*; stupid sleep with unconsciousness: *Opium.*

Restless, stupefying dull sleep, with constant tossing about: *Pulsat.*

Lethargic slumbering, with murmurs and snoring: *Rhus tox.*

Comatose sleep: *Baptis. Colchic.*; comatose slumbering full of troubled and intermittent dreams: *Rhus tox.*; — with anxious delirium: *Bryon.*

The night seems long and tedious, with comatose slumbering, and dreams full of bustle and hurry: *Nux vom.*

Unconquerable coma vigil: *Coccul. Hyosc.*; with indistinct muttering: *Opium*; alternating at night with coma somnolentum, delirium, hot skin, and stupidity: *Opium*; with frequent starts as if from fright, and one eye open and the other closed or half open: *Veratr.*

Constant somnolence, may be roused, but not to full consciousness: *Helleb.*

Slumbers day and night with murmuring delirium: *Phos. ac.*

**Coma**: *Coccul. Veratr. alb.*; constant profound —: *Psorin.*; profound comatose sleep with snoring (see

Opium); sometimes opening the eyes, without moving, with wild look: *Bellad.*; profoundest —, lying silent, immovable, insensible; a dreamy state, with drowsiness, and falling of the eyelids, restless sleep at night: *Nux mosch.*; irresistible sleep and complete —, with insensibility; heat, pulse, and respiration as in health: *Opium.*

When spoken to, answers correctly, but soon relapses into stupor again: *Hyosc.*

**Sopor**: *Arsen. Laches. Lycop. Opium. Phosphor. Rhus tox.*; deep: *Coccul.*, could not be awakened: *Opium*; — and delirium: *Lycop.*; — and stupefaction: *Phosphor.*; stupid, soporous comatose state: *Arnic.*, with staring eyes: *Opium*; febris soporosa of children; heat, chewing motions, turning the occiput or putting their hands there: *Carb. veg.* 2$^{c}$. *Opium*; — with open eyes: *Lycop.*; fevers with soporous condition, dry black lips and tongue, and open mouth: *Phosphor.*; —, lying on the back, tongue heavy, face sunken, lower jaw hanging down, pulse small, weak, and frequent: *Laches.*; — threatens to terminate in paralysis of the brain: *Opium.*

Lies at night in bed without consciousness, with groans, cold sweat on the forehead, followed by weakness: *Bryon.*

Great dullness of mind, with bodily weakness: *Laches.*

Complete insensibility: *Laches.*

Great anxiety and fearfulness drives from the bed, springs out and calls for help, on account of indescribable feeling of distress: *Rhus tox.*

He springs out of bed delirious, in evening slumber: *Nux vom.*

Delirious phantasies, on lying down: *Nux vom.*

Muttering and phantasies in sleep: *Nitr. sp. d.*

Much muttering during the evening fever: *Laches.*; unintelligible muttering: *Pulsat.*; whimpering, at three o'clock: *Bryon.*; moaning and whimpering: *Nux vom.*

Sleep with outcries: *Bryon. Hyosc. Lycop. Phosphor.*, wakening: *Stramon.*

Delirium at night: *Bryon. Laches. Pulsat.*

Insensibility of the organs of touch, sight, and smell: *Opium.*

Heaviness of the head, with dullness like lead in the occiput, with vertigo: *Laches.*

Distorted open eyes: *Opium*; half open: *Opium. Sulphur.*

Nose bleed at night: *Bryon. Mercur. Rhus tox. Veratr.*, after midnight: *Rhus tox.*

Jerkings of the face: *Bryon.*

Face red, swollen, with injected conjunctiva: *Bellad.*; with dark brown face: *Stramon.*

Distortion of the mouth, or movements like chewing: *Bryon.*

Open mouth: *Rhus tox.*; half open mouth, red, puffy face: *Opium*; hanging of lower jaw: *Opium.*

Thirst, with frequent drinking: *Bryon.*

Involuntary stools: *Bryon.*

Involuntary evacuations of fæces and urine: *Arnic.*

Suffocating night mare: *Opium.*

Blowing, whistling expiration through the nose: *Nux vom.*; with very deep drawn respiration:

*Stramon.* ; loud blowing with inhalation and exhalation : *Arnic.*

Respiration slow, heavy, or even intermittent: *Opium.*

Very short breath : *Rhus tox.*, with rapid pulse, oppressed and anxious respiration : *Opium.*

Snoring inspiration, as if the posterior nares were contracted, before midnight : *Nux vom.*

Suffocating snoring with the inspiration : *Bryon.*

Snoring in sleep : *Sulphur*, and murmuring, and picking at the bed-clothes : *Rhus tox.*

Whimpering, sighing, and moaning : *Opium* ; whimpering, loud talking, loud blowing inhalation and expirations : *Arnic.*

Pulse slow, suppressed : *Opium* ; first rapid and strong, then weak and intermitting : *Opium.*

PULSE ; very small, rapid, and intermittent ; weak, irregular, often intermitting ; small, rapid, or hardly perceptible : *Stramon.* ; rapid and weak : *Opium.*

Paralytic sensation in all the limbs : *Rhus tox.*

Constant sliding down in bed, with groaning and moaning in sleep, and muttering and unconsciousness whilst awake : *Mur. ac.* See Positions.

Sighing and jerks in sleep, which awake the patient : *Bellad.*

Jerking of limbs, muscles of the face, and corners of mouth : *Opium.*

Constant slumber with picking : *Hyosc.* ; touching surrounding objects, with half-opened eyes : *Opium.*

Shuddering from the slightest current of air : | *Bellad.* | *Selen.*

Throwing off the bed-covering, because of heat, most in the palms of the hands: *Pulsat.*; he casts off the clothes because they are too tight, or too warm, yet he shivers as soon as he is uncovered: *Pulsat.*

Dry heat: *Bryon.*; with pain in the part on which he has lain, as if the place had been too hard: *Phosphor.*; with dryness of the mouth, which impels him to drink: *Phosphor.*

Burning —, both internal and external: *Hyosc.*, on palms of hands: *Pulsat.*

**Sweat**; quiet sleep, with profuse sweat: *Hyosc.*; night sweats: *Hyosc.*; — greasy, stiffens the linen, stains it yellow: *Laches.*, or is offensive: *Mercur.*; copious —, with itching of a miliary eruption: *Opium.*

**Dreams**; troublesome: *Baptis. Ignat.*; full of phantasies and dreams in long continuing slumber: *Pulsat.*; extravagant —: *Nux vom.*; delirious, anxious —: *Arnic. Hyosc.*, and heaviness from the evening into the night; — which much affect the body: *Arnic.*; wakes from — with fright and outcries: *Bryon.*; frightful —: *Arsen. Bellad.*; is kept awake for fear of horrible visions: *Bryon.*; — angry, as if bitten by a dog and he cannot escape; as if he were hunted; of robbers, with frightened waking, and a fixed idea that the dream is true: *Veratr.*; — frightful: *Arnic.*, as if he fell from a height, or as if he were not in his own home, and talking of distant villages; of shootings; of street-robbers: *Mercur.*; of animals biting, with outcries, and waking with anxiety: *Phosphor.*; as if cut and hacked by soldiers, with desire to escape: *Bryon.*; — anxious every night: *Sulphur*; — full of images: *Nux vom.*; with subsequent vomiting of green, tenacious slime: *Veratr.*; sleep full of —, and restless before midnight; irrational talk in his sleep like delirious dreams: *Sulphur*; frightful —, and frequent waking, and never that state of quiet, profound coma: *Rhus tox.*; restless, with constant, heavy —: *Secal.*; dreaming at night: *Bryon. Phosphor. Rhus tox.*; restless sleep, with frightful — of being drowned: *Veratr.*

**Restless**; from abdominal symptoms: *Nux mosch.*; — tossing at night in bed: *Sulphur*; — tossing, as if from great heat: *Pulsat.*; only snatches of sleep: *Camphor*; sleepless, wandering mind: *Baptis.*; intolerable —, anxiety and discomfort at night in bed, with sleeplessness: *Mercur.*; with outcries and loud laughing: *Lycop.*; — with groaning and moaning, and frequent movements of the mouth, like chewing: *Bryon.*; — disturbed sleep with frequent turning, and throwing off the bed covering: *Rhus tox.*; — throwing about in bed: *Pulsat.*; — at night; continual restless sleep and vomiting: *Bellad.*

**Sleeplessness**; *Phosphor.*, with great restlessness and tossing about the bed: *Arsen.*; — from same idea rousing him from slumber: *Calc. ostr.*; — from nervous excitability: *Hyosc.*; utter — from overactivity of mind: *Calc. ostr.*; — with strong desire to sleep: *Bellad.*; —, stupid, with phantasies of dragons, skeletons, horrible spirits, ghosts; in a state of half sleeping and waking: *Opium*; frightful imaginations prevent his sleeping in the evening: *Mercur.*; — with restlessness and delirium, or incomplete visions and phantasies: *Opium*; — on account of various visions as soon as he falls in a doze: *Ignat. Calc. ostr.*;

— or constant sleep with muttering: *Hyosc.*; — with carbo-nitrogenoid constitutions: *Sulphur.*

**Waking**; half awake; phantasies of headless bodies and dead acquaintances: *Nux vom.*; waking suddenly from sleep with start and fright: *Bellad.*; — frequently as if from fright, or from wakefulness, with much tossing: *Mercur.*; — from each sleep with fright as if from a terrifying dream: *Sulphur*; — in fright from the least noise: *Nux vom.*; — frequently from sensation of heat: *Phosphor.*; — insensible: *Cinchon.*; stupefied as if drunk, dizzy, staggering: *Phosphor.*; on going to sit up, giddy, has to lie down again: *Opium.*

When awaking, exceedingly cross, irritable, scolding, screaming, behaving disagreeably: *Lycop.*

After waking, anxious phantasies, as from ghosts, or his employments, from which he cannot at once free himself: *Sulphur.*

Waking delirium: *Bryon.*; with loud outcries: *Stramon.*; that which he has dreamed, seems to him a reality: *Sulphur. Natr. mur.*

On waking, whirling in the head, by which sleep is made more distressing, than pleasant: *Mercur.*; — weak, sluggish, drowsy lassitude: *Bryon.*

**After sleep**; much exhausted: *Secale*; after morbid sleep, stammering; difficulty of moving the tongue; nausea: *Opium*, — aggravation of all the symptoms: *Laches.*

Sleeplike state, from which he emerges with consciousness and speech: *Mercur.*; sleeps all the time, awakens conscious, but while awake taciturn, apathetic: *Phosph. ac.*

**Sleep unrefreshing,** and full of dreams: *Arnic.*; after sleep weary as before: *Bryon. Nux nom.*; feels after light sleep as if he had not slept at all: *Pulsat.*; as if he had too little sleep: *Veratr.*

Loss or interruption of sleep is followed by great loss of strength: **||** *Coccul.*

**At night**; diarrhœa worse: *Pulsat.*; after midnight dryness and thirst: *Rhus tox.*; at three A. M. nose-bleed: *Bryon.*, anxiety: **||** *Kali carb.*; all symptoms worse about and soon after midnight: **|** *Arsen.*; after midnight, delirium: *Kali carb.*; worse after than before midnight: *Rhus tox.*; at night, delirium: *Bryon.*

**Morning**; remission: *Rhus tox.*; delirium: *Bryon.*; dry tongue: *Opium. Sulphur*; sudamina appear: *Mercur.*

AFTERNOON; dryness and thirst: *Rhus tox.*; sleepy: *Bellad.*

ALL DAY; heavy and weak: *Sulphur.*

EVENING; aggravation: *Bryon.*; frightful phantasies: *Cinchon.*; delirious: *Bryon.*; bleeding from the nose: *Phosphor.*; pale, sick aspect: *Phosphor.*; dry mouth, without thirst: *Nux mosch.*; fever and fainting: *Laches.*

Symptoms recur at the same hour: *Moschus.*

**Cold and warmth**; washing in cold water relieves: *Fluor. ac.*; heat aggravates all the symptoms: *Jodium*, relieves icy coldness of body: *Lachnanth.*; weariness as if from overheated air: *Veratr.*; external warmth is unbearable; warm sensation as if in an overheated room, or as if hot air were blowing on one, which excites headache: *Pulsat.*

External heat is intolerable, causing a sense of heat with distress; uncovering, however, is followed immediately by a chill, at the early stage: *Pulsat.*

Uncover themselves: *Bellad.*; desire to throw off the covers: *Fluor. ac.*; would not be covered up: *Secale*; desire to be covered, with dry heat or with sweat: *Rhus tox.*

Immoderate sensibility to the open air: *Nux vom. Selen.*

TEMPERATURE; sunken: *Coccul.*, collapse with it: *Camphor*; quickly decreasing, most on limbs: *Camphor.*

**Chilliness and coldness**; coldness, and no other symptoms: *Lycop.*; — of the whole body, especially the feet and hands, would not be covered: *Secale*; with cold sweat upon the hands, feet, and face: *Veratr. vir.*; clammy cold skin: *Camphor*; covered with clammy sweat: *Rhus tox.*; skin cool, heat internal, face flushes: *Jodium.*; sweat on one side, coldness of the other: *Pulsat.*; shiverings: *Baptis.*; — and heaviness of body: *Pulsat.*; violent chill each time after eating or drinking: *Tarax.*; — all day: *Baptis.*; — with soreness of the whole body: *Baptis.*; — with sighing: *Ipec.*; begins with chills, headache and backache; pain in the limbs: *Baptis.*; chill and fever simultaneous, with soreness of the flesh: *Baptis.*; chills alternating with heat; moist skin: *Baptis.*

**Fever and heat**; temperature of the body not high, and even, all over: *Phosph. ac.*; burning heat: *Arsen.*; increased warmth: *Stramon.*; moderate warmth of skin: *Helleb.*; skin dry and warm: *Mur.*

*ac.*, and clammy: *Phosph. ac.*; dry skin, burning hot: *Phosph. ac.*; biting hot: *Bellad.*; burning of the skin while laying the hand on any part of the body: *Hyosc.*; local heat: *Calc. ostr.*; heat only on one side: *Pulsat.*; heat at night: *Baptis.*; heat worse in the afternoon: *Laches.*; heat and delirium on going to sleep: *Ginseng*; burning fever with somnolency and delirium: *Lachnanth.*; sensation of heat in body, most in head: *Bryon. Rhus tox.*; fever with circumscribed redness of the cheeks in the evening: *Arsen.*, and brilliant eyes: *Lachnanth.*; heat intense, with raw mouth: *Arum triph.*; heat and thirst: *Bryon. Rhus tox.*; heat without desire to drink: *Hyosc.*; burning and vomiting: *Chamom.*; anxious and restless, with burning, as if hot water were flowing through the veins, or with throbbing in the head, and desire to throw off the bed-covers: *Arsen.*; trunk hot, but limbs cold: *Colchic.*; skin normal temperature: *Helleb.*; mostly dry with increased temperature: *Mur. ac.*; hot and dry: *Gelsem.*; dry skin burns: *Pulsat.*; dry or clammy: *Phosph. ac.*; heat all over with sweating, most in the evening, in bed, or during the day, with pale face: *Veratr.*; skin dry: *Colchic.*, and burning: *Apis*; like parchment and pungent hot: *Arsen.*

**Sweat**; cold: *Carb. veg. Veratr.*; — on whole body: *Veratr.*; — all over: *Camphor*; — on forehead: *Coccul.*; — on forehead only: *Veratr.*; — in face: *Bellad.*; — mostly on face and forehead: *Secale*; — only on head and trunk: *Veratr.*; — on hands: *Coccul.*; — on hands, feet, and face: *Carb. veg.*; — with trembling of the whole body: *Pulsat.*; — cold and clammy: *Arsen.*; hot — on the face: *Rhus tox.*; inclination to perspire, with very hot skin: *Bellad.*; disposed to much —: *Carb. veg.*; — profuse, whole body bathed in it: (*Stramon.*); — general and profuse: *Secale*; profuse —: *Phosph. ac.*; and stupor: *Kali carb.*; — with desire to be magnetized: *Silic.*; — clammy, partial: *Apis*; — sticky, constant: *Phosph. ac.*, and cold: *Arsen. Phosph. ac. Secale. Veratr.*; frequent —: *Baptis.*; copious, debilitating —: *Mercur.*; critical sweat on forehead and face: *Baptis.*; acid — at night, preceded by thirst, with pressing drawing in the head toward the end of the sweating, followed by confusion of the head: *Bryon.*

Sour sweat at noon, and sour diarrhœa in daytime: *Rhus tox.*; — most in afternoon and in upper part of body: *Fluor. ac.*; — makes patient thirsty: *Jodium*; — without thirst: *Rhus tox. Sambuc. Sepia. Veratr. alb.*

Fetid sweat: *Baptis.*; offensive and sour: *Arsen.*, and breath offensive: *Arnic.*; a cadaverous smell scents the whole atmosphere: *Arsen.*

Great disposition to sweat: *Colchic.*; during the day: *Pulsat.*; gentle sweating, during which he wishes to be covered: *Rhus tox.*; anxious sweat, preventing sleep, with sighing, short cough, and pressure in chest: *Bryon.*

In sleep: *Cinchon.*, in bed, in the morning: *Rhus tox.*; nocturnal, also continued, mostly in a long sleep: *Veratr.*

With great weakness and stupidity: *Hyosc.*

From the head to the epigastrium: *Secale.*

On one side; on the right side of the face, or one side of body, either the right or left: *Pulsat.*

Over the whole body, except the head: *Rhus tox.*; moist skin all over: *Bryon.*

With the rash: *Stramon.*

Suppressed sweat: *Colchic.*

Sweat did not relieve: *Laches. Mercur. Phosphor.*; children, with great dryness followed by hot sweat, without relief; not Mercur., but *Stramon.*

Long lasting sweat in reconvalescence: *Psorin.*

Gets bedsores on parts sweating: *Fluor. ac.*

**Directions**; complaints, which go from above downwards: *Selen.*; commencing below and spreading upwards: *Fluor. ac. Guaco.*

**Periodicity**; fever comes on by short periods: *Bellad. Calcar.*

**Sensations**; neuralgic pains leave suddenly: *Phosphor.*; exalted irritability of all the organs: *Bellad.*; sensation as of lead in the veins, worse when sitting: *Mercur.*; stiffness (in the beginning): *Bryon.*

Rheumatic pains: | *Bryon.* | *Camphor.* | *Rhus tox.* The more rheumatic pains in the beginning, the more we may expect abdominal localization. The patient will call pains rheumatic, but they are nervous and decrease when thinking about it. *Camphor.* JAHR.

Dull, pressive, or stitching, tearing pains, worse from motion and opening the eyes: *Bryon.*

Shooting pains in one or the other part of body, whilst the part is at rest: *Rhus tox.*

Pains throbbing: *Bryon.*

**Tissues**; throbbing in veins; they enlarge here and there: *Jodium*; bleedings from the beginning:

*Nitr. ac.*; hemorrhages: *Alum. p. s. Arsen. Bryon. Carb. veg. Cinchon. Nitr. ac. Phosphor. Rhus tox. Sulph. ac.*

Exhaustion from loss of fluids: *Cinchon.*; anæmia: *Chin. sulph.*

Little or no affection of mucous membranes of intestines: *Helleb. Mur. ac.*

Acrid ichorous discharge from nose, excoriating mucous membranes, alæ nasi, upper lip, making them raw and sore: *Arum triph.*

Soreness in all the bones: *Pulsat.*, aching in bones and back: *Baptis.*

Want of firmness in all the joints: *Bryon.*; the joints seem too weak to carry the body: *Carb. veg.*; weakness of joints: *Phosphor.*

Relaxation of the muscles with exhaustion from the slightest exertion: *Laches.*; muscles relaxed, with waxy skin: *Veratr.*

Trifling loss of flesh: *Helleb.*

Emaciation: *Arsen. Colchic. Mercur. Opium*; extreme: *Nuphar*; rapid: *Secale.*

Fetor of breath, sweat, urine, and fecal discharges: *Baptis.*

Putrid decomposition of fluids: *Rhus tox.*; great prostration with disposition in the fluids to decompose: *Baptis.*; no signs of putrid dissolution: *Helleb.*

**Touch**; sensibility of the surface: *Colchic. Hamam.*; bed-covering causes pain: *Laches. Sulphur*; when touched, pain in hypogastrium, abdomen, and loins: *Phosphor.*

Sensitive to pressure on stomach: *Arsen. Phosphor.*, on abdomen: *Phosphor.*

Dullness and bruised pains in the limbs : *Laches.*; with sensation as if bruised, lasting the whole night: *Pulsat.* ; bruised soreness, every position feels too hard : *Arnic.*

Bluish red spots on the parts which the patient lies upon: *Phosph. ac.*

DECUBITUS ; on the sacrum and trochanter: *Zincum*; bed-sores: *Arnic. Arsen.* | *Carb. veg. Cinchon.* || *Fluor. ac. Moschus. Nux vom. Phosph. ac.*, gangrenous, like sphacelus: *Carb. veg.*

**Skin** ; small transparent vesicles filled with watery fluid, appearing in the morning on various parts of the body (Sudamina): *Mercur.*

White rash : *Apis. Laches. Mur. ac.* ; in beginning: *Bryon.* ; white miliary rash : *Arsen.* ; with anxiety in the region of the heart : *Bryon.*

Red rash : *Phosphor.* ; insufficient : *Lycop.* ; scattered : *Phosph. ac.* ; red miliary spots on chest and back : *Cinchon.* ; after stupefaction of the head, dullness of vision, beclouding of all the senses, the rash appears on the back, with sweating : *Stramon.*

Repeated breaking out with short, frequent breathing: *Arsen.*

Miliaria purulenta with anasarca: *Sulphur.*

Miliary rash : *Rhus tox.*, on the trunk : *Phosphor.*, about the neck, then back, then chest, gradually all over, lastly upon the feet : *Phosph. ac.*

Lenticular red spots with small vesicles in the centre : *Rhus tox.*

Numerous roseola spots : *Rhus tox.*

Exanthem delayed, and on the fourteenth day fever again rising, with new symptoms of cerebral irritation, twitchings, delirium, frightful visions, or great anguish (whether diarrhœa is present or not): *Calc. ostr.*

Bluish spots: *Nux mosch.*; extravasation under the epidermis: *Arsen.*

Ecchymosis: *Carb. veg. Phosph. ac.*; on trunk: *Arnic. Phosphor.*; ecchymotic spots here and there: *Carb. veg.*

Large purple spots on the body, particularly on the feet: *Secale.*

Red spots like flea-bites, or blood stains, or petechiæ, on chest, abdomen, face, and neck: *Bellad.*

An eruption like flea-bites: *Pulsat.*

Petechiæ: *Arsen. Bryon. Secale. Rhus tox. Stramon.*; small shining, star-shaped, on the face, throat, and chest: *Stramon.*; with great weakness, even to the loss of all strength: *Rhus tox.*

After sudamina and petechiæ have appeared, and no diarrhœa set in: *Bryon.*

Icteroid color of the skin: *Mercur.*, waxy, pale, pale as wax: *Rhus tox.* See Face.

Jaundice with diarrhœa: *Leptandr.*

Skin cool, bluish: *Carb. veg.*; relaxed pale: *Phosph. ac.*; wrinkled: *Veratr.*; small, red, itching spots here and there on the body: *Opium.*

Ulcerations: *Baptis.*; boils: *Mercur.*

Decubitus. See Touch.

**Constitutions**; at the very onset all further progress may be cut off, in persons inclined to grow fat, by: *Calc. ostr.*; weak, debilitated individuals, old age or children: *Arsen.*

In cholera time: *Cuprum. Sulphur.* | *Veratr.*

**Other drugs**; after abuse of Calomel: *Nitr. ac.*; after Opium: *Helleb.*; after abuse of Chloral: *Nitr. ac.*, — Mercur.: *Sulphur*; — Calcar.: *Lycop.*; — Bryon.: *Mur. ac.*

*Camphor* in cases like *Carb. veg.*, but symptoms more rapid.

*Bryon.* and *Rhus tox.* rarely in alternation, but *Rhus tox.* and *Nux vom. Bryon.* and *Pulsat.* (Bœnninghausen) of course not without being indicated by the changing symptoms.

When *Rhus tox.* or *Bryon.* are insufficient in catarrhal or pneumonic symptoms, compare *Phosphor.*

If *Nitr. ac.* be insufficient in bleeding from anus, the symptoms may have been more for *Mur. ac.*, also *Arsen.* or *Phosphor.* See Gross's Mat. Med. Comparativa. *Alum. p. s.* may be the only resort. See Mat. Med.

When *Rhus tox.* or *Arsen.* are insufficient in cases of great prostration, compare *Mur. ac.*, after it *Carb. veg.*

In hepatic affections, after *Byron. Laches.* and *Lycop.* had failed, *Mercur.* was given with success.

### MODEL CURES.

In the war-typhus of 1813 to 1814, where in the *first stage* of the fever *Rhus tox.* or *Bryon.* (never in alternation), in the *second stage Hyosc.*, in the twelfth centesimal, answered so well, that from 183 cases only one, an old person, died, there sometimes occurred a *third stage*, a sort of lethargy of the sensorium, a kind of half paralysis of the mental organs. The patient remained lying indolently without sleeping or speaking; he scarcely answered whatever might have been done to induce him to do so; he appeared to hear, without understanding what was said or without allowing it to make any impression on him (the few words he said, he whispered; but not irrelevantly); he appeared to feel almost nothing and to be almost immovable, and yet not quite paralyzed. In this case, Sweet Spirit of Nitre was useful. It must be so old that it no longer reddens the cork. One drop is to be shaken up with an ounce of water and given by teaspoonfuls so as to be consumed in the four and twenty hours. In the course of a few days this state passed into health and activity. SAMUEL HAHNEMANN'S Lesser Writings, p. 634.

An extreme case. Great thirst, but fluids caused pain and gurgled through the intestines, making a noise as in an empty cask. *Hydroc. ac.* HAYNEL.

In prevailing fever, with sopor. No complaint except thirst. On lifting the head dullness and vertigo, had to lie down. *Opium.* H. GROSS.

The patient lies in an unconscious state, with both arms lying stretched out by the side of the body; now and then starts up suddenly as if mad, and strikes about him; with screaming, tossing to and fro, or even clonic spasms. All in consequence of erosions and ulcers of inner organs—*f. i.*, the intestinal canal, or an inflammatory condition of the membranes of the brain: *Canthar.* H. GROSS.

Typhoid fever with the greatest indifference, putrid breath, and red spots like suggulations on the body: *Arnica*. C. HG.

An elderly man given up with a typhoid fever, suddenly took a change for the better after *Plumbum* 30, not only because many of his (altogether objective) symptoms were similar to it, but he had been an opium-eater for years. After consciousness came back he turned yellow all over, his appetite came back, and he recovered fully. C. HG.

Relapse in third week ; sudden stupor and speechlessness with a brown parched tongue as hard as a board, teeth and gums covered with a brown mucus, on the nose dried-up hard crusts. *Rhus tox.* in water, a teaspoonful every two or three hours; followed by recovery within three days. JAHR.

Malignant ship-fever; ninth day; lying on his back unconscious ; eyes wide open, glaring, fixed on the ceiling; pupils dilated; cheeks red and hot; mouth wide open; jaw hanging down; lips and tongue dry, black, cracked ; picking of bed-coverings ; pulse 200. Involuntary urination at night, on the sheets a large deposit of red sand, like brick-dust. *Lycop.* $2^c$ in half a tumbler of water a spoonful every two hours; after six or eight hours, eyes and mouth closed, sleep and sweat and full recovery. A. LIPPE.

Violent delirium with severe pains in the limbs, great weakness, dry tongue (red or black); dry, brown, or black lips; heat and redness of the cheeks, carphologia, pulse quick and small, lethargic slumbering, with murmurs and snoring: *Rhus tox.* WELLS.

Large purple spots on the body, particularly on the feet; body cold, especially the feet and hands, and would not be covered up; cold perspiration, mostly on the face and forehead; copious vomiting of greatly degenerated thick black bile, mixed with tenacious bilious mucus; this bilious matter was badly mixed with black bile, mucus, degenerate substance, etc.—it was a *horrible* mixture; would vomit about a quart at a time: *Secale corn.* high. GUERNSEY AND LIPPE.

In a case of ship-fever, apparently sinking, quick fluttering pulse, could hardly move. *Iris vers.* GUERNSEY.

Tongue dry and brown. *Thirst for large quantities.* Delirium at night. *Desire to get out of bed and go home. Talking about business of the past few weeks. Bryon.* $2^c$. $2^m$. J. B. BELL.

Much headache. Dreaming and *talking in his sleep about business of the past few weeks.* Thinks about it in daytime. *Bryon.* $2^c$. $2^m$. J. B. BELL.

*Violent subsultus tendinum. When spoken to answers correctly, but quickly relapses into stupor. Hyosc.* $2^c$. in twenty-four hours. A young man, third week of typhoid fever. J. B. BELL.

Third week violent and almost constant cough; expectorates bloody, frothy mucus. Stupor, profuse sweat. Involuntary stool. Pulse 120 to 140. Tongue clean on tip, pointed. *Pale watery swelling in a little bag over the eyes. Kali carb.* $2^c$. J. B. BELL.

Entire unconsciousness like a deep sleep, mouth open and filled with a slimy mucus. Deathly appearance. Breathing rapid and labored. Pulse feeble and rapid. *Whole body bathed in profuse perspiration which did not relieve.* Urine passed unconsciously in bed, leaving a whitish sediment on the parts, legs, and sheets, where it passed. *Merc sol.* $6^m$. J. B. Bell.

